# Bioevaluation of *Pheretima vulgaris* Antithrombotic Extract, *PvQ*, and Isolation, Identification of Six Novel *PvQ*-Derived Fibrinolytic Proteases

**DOI:** 10.3390/molecules26164946

**Published:** 2021-08-16

**Authors:** Wanqing Yang, Wenjie Wang, Yunnan Ma, Qilin Yang, Pengyue Li, Shouying Du

**Affiliations:** School of Chinese Materia Medica, Beijing University of Chinese Medicine, Chaoyang District, Beijing 100029, China or 20180941263@bucm.edu.cn (W.Y.); 20180941319@bucm.edu.cn (W.W.); 20180935073@bucm.edu.cn (Y.M.); 20200935146@bucm.edu.cn (Q.Y.); pengyuelee@bucm.edu.cn (P.L.)

**Keywords:** *Pheretima vulgaris*, isolation, fibrinolytic activity, structural identification, LC-MS/MS, Edman degradation, gene cloning

## Abstract

Thrombosis is a disease that seriously endangers human health, with a high rate of mortality and disability. However, current treatments with thrombolytic drugs (such as recombinant tissue-plasminogen activator) and the oral anticoagulants (such as dabigatran and rivaroxaban) are reported to have a tendency of major or life-threatening bleeding, such as intracranial hemorrhage or massive gastrointestinal bleed with non-specific antidotes. In contrast, lumbrokinase is very specific to fibrin as a substrate and does not cause excessive bleeding. It can dissolve the fibrin by itself or convert plasminogen to plasmin by inducing endogenous t-PA activity to dissolve fibrin clots. Therefore, searching for potentially new therapeutic molecules from earthworms is significant. In this study, we first collected a strong fibrinolytic extract (*PvQ*) from the total protein of the *Pheretima vulgaris* with AKTA pure protein purification systems; its fibrinolytic bioactivity was verified by the fibrin plate assay and zebrafish thrombotic model of vascular damage. Furthermore, according to the cell culture model of human umbilical vein endothelial cells (HUVECs), the *PvQ* was proven to exhibit the ability to promote the secretion of tissue-type plasminogen activator (t-PA), which further illustrated that it has an indirect thrombolytic effect. Subsequently, extensive chromatographic techniques were applied to reveal the material basis of the extract. Fortunately, six novel earthworm fibrinolytic enzymes were obtained from the *PvQ*, and the primary sequences of those functional proteins were determined by LC-MS/MStranscriptome cross-identification and the Edman degradation assay. The secondary structures of these six fibrinolytic enzymes were determined by circular dichroism spectroscopy and the three-dimensional structures of these proteases were predicted by MODELLER 9.23 based on multi-template modelling. In addition, those six genes encoding blood clot-dissolving proteins were cloned from *P*. *vulgaris* by RT-PCR amplification, which further determined the accuracy of proteins primary sequences identifications and laid the foundation for subsequent heterologous expression.

## 1. Introduction

As commonly known, thrombosis is a common underlying factor for the occurrence of cerebro-cardiovascular diseases, such as stroke, heart attacks, and pulmonary embolisms [1,2]. Thrombotic diseases present a significant risk of mortality and disability, which has seriously affected public health [3]. Dissolving the thrombus is essential in the treatment of thrombotic diseases. However, the current drug used for thrombolysis exhibits various side-effects, the most dangerous being life-threatening bleeding [4]. Since bioprospecting introduced the promise of additional sources of bioeffective products with fewer side-effects, it has become possible to explore complementary and alternative therapies. As an alternative therapy, medicinal animal preparations from leeches, snakes, and earthworms have appealed great attention in the half past centuries [2]. Among these preparations, lumbrokinase from earthworms has been used as a fibrinolytic medicine, and oral lumbrokinase preparations have been extensively applied to support circulatory health and treat blood diseases in many countries [5]. Additionally, the earthworm fibrinolytic enzyme has shown broad prospects in the development of novel effective clot-dissolving drugs.

*Pheretima vulgaris*, a medicinal animal, is recorded in the current issue of Chinese Pharmacopoeia. It has been used as a traditional medicine in China, Japan, and southeast Asian countries for centuries, and possesses outstanding bioactivities in terms of fibrinolysis and anti-thrombosis [6]. Although various fibrinolytic extracts have been discovered from different earthworm species, such as *Eisenia fetida* [7,8,9], *Lumbricus rubellus* [10,11], among others [12,13,14], since the first report of lumbrokinase by Mihara’s team in 1991 [15], few fibrinolytic activity studies related to *P. vulgaris* are reported. Moreover, the complexity of medicinal animal compositions has led to the previous reports being either evaluations of the activity of crude extracts [11,16] or vague descriptions of mixtures [17], with few reporting specifically on functional monomers. Additionally, 42 genes encoding lumbrokinase are publicly available at NCBI GenBank, but most of these genes are from *Eisenia fetida* and *Lumbricus rubellus*. Until now, fibrinolytic genes from *P. vulgaris* have not been reported. Therefore, further research into the nature and function of medicinal compounds derived from *P. vulgaris* is needed.

The purposes of this study are to find highly fibrinolytic ingredients, and further isolate and identify functional proteins through bottom-up proteomic analysis and the Edman degradation assay. Briefly, a group of fibrinolytic proteins, named *PvQ*, from *P. vulgaris* were collected, and their bioactivity was further evaluated by the fibrin plate method and zebrafish thrombotic model of vascular damage. All the results showed that *PvQ* possesses significant fibrinolytic activity both in vivo and in vitro. In terms of indirect thrombolysis, the *PvQ* can also promote the release of t-PA on endothelial cells. Then, in order to reveal the material basis of *PvQ*, a further separation was applied via AKTA pure combined with extensive chromatographic techniques, including ion exchange, sizes-exclusion and hydrophobic chromatography. Six novel *P*. *vulgaris*-derived fibrinolytic proteins, named *Pv*I–*Pv*VI, were obtained from *PvQ*. Additionally, based on the primary structure of these proteins, their secondary structures were identified by CD spectroscopy and their 3D structures were predicted by homology modelling through MODELLER 9.23. Thereafter, genes encoding the six proteins were cloned successfully, the results provided the research basis for subsequent heterologous expression. In sum, *PvQ* has shown therapeutic prospect for dissolving clots. Additionally, its active components, *Pv*I–*Pv*VI, can be applied as potential fibrinolytic molecules against thrombosis or be used as a pro-drug to develop novel recombinant drugs through genetic engineering and monoclonal antibody technology.

## 2. Results

### 2.1. Enrichment of Active Extract

**Figure 1:**
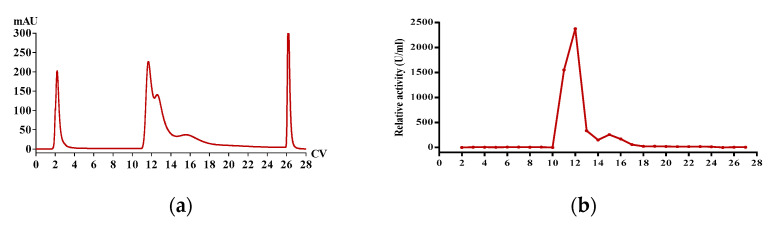
(**a**) HiTrap Q HP chromatogram of *P. vulgaris* crude extract and (**b**) the fibrogenesis inhibition activity of 27 eluted fractions.

The fibrinolytic extract from *P*. *vulgaris*, named *PvQ*, was gathered from crude earthworm extract by the prepacked ion-exchange chromatography column—HiTrap Q HP—with a stepwise gradient elution of NaCl (from 0.1 to 1 M) at pH 7.8 on the AKTA pure and three eluted peaks were collected (Figure 1a). Thereafter, the activity of each peak was evaluated through a Fibg-TT assay and the result showed that peak 2 exhibits a significant fibrogenesis-inhibiting effect (Figure 1b). Consequently, the constituents of peak 2 were considered as the functional extract.

### 2.2. Determination of Fibrinolytic Activity

**Figure 2:**
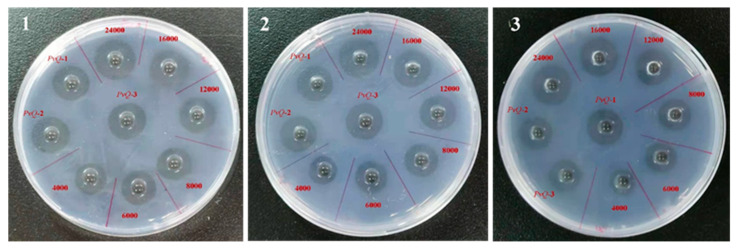
The fibrin-plate assay of *PvQ* and lumbrokinase.

As evident from the formation of the clear lysis zone on the fibrin plate (Figure 2), *PvQ* degraded fibrin into soluble peptides, which revealed its fibrinolytic activity. After incubation for 18 h, the specific activity [18] of *PvQ* relative to lumbrokinase was calculated according to the measured diameter results, as shown in Table 1. Furthermore, the specific activity of *PvQ* (247 U/μg) was about twice higher than that of lumbrokinase (115 U/μg). This indicates that *PvQ* possesses an excellent and long-acting fibrin-hydrolysis activity in vitro. Experiments were repeated on three dishes for each sample.

### 2.3. Evaluation of Thrombolytic Activity In Vivo

**Figure 3:**
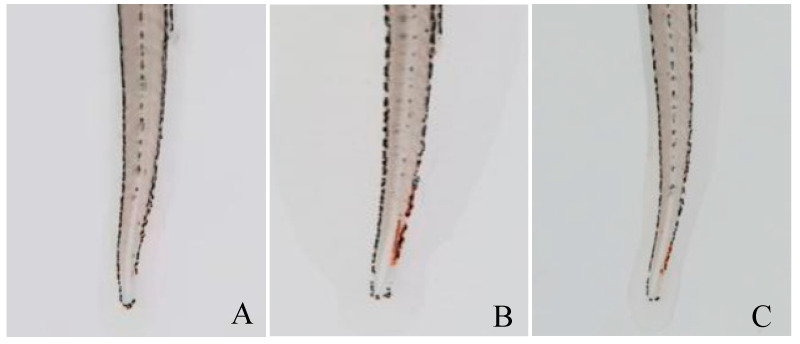
The staining of erythrocytes in the tail vein of zebrafish in (**A**) the normal group, (**B**) the model group and (**C**) the treatment group.

**Figure 4:**
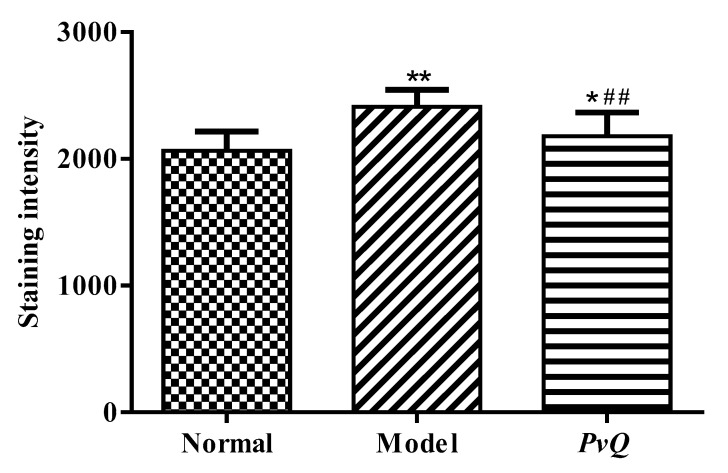
The staining intensity of tail vein erythrocytes. Data are expressed as mean ± SD (*n* = 18). * *p* ≤ 0.05, ** *p* ≤ 0.01 compared to the normal group; ^##^ *p* ≤ 0.01 compared to the model group.

The antithrombotic effects of *PvQ* in vivo were further evaluated using the zebrafish thrombus model of epinephrine hydrochloride-induced vascular endothelial injury [19,20]. After administration, the heart rate and arterial pulse frequency of zebrafish were increased, which accelerated the blood circulation in veins; thrombosis developed rapidly in the tail vein. Therefore, the successful modelling not only resulted in the formation of venous thrombosis, but also accompanied by the increased heart rate and arterial pulse. Thereafter, the tail vein thrombosis of zebrafish was directly observed and photographed under a microscope, as shown in Figure 3. Obviously, zebrafish had no thrombosis in the normal group (A), while zebrafish in the model group (B) formed severe thrombosis, and the staining intensity of tail vein erythrocytes in the *PvQ* group (C) was significantly reduced, indicating that most of blood clots were hydrolyzed by *PvQ*. Furthermore, the quantitative analysis results showed that *PvQ* has a significant in vivo fibrinolytic effect with an inhibitory rate of 67.3% at a dosage of 10 ng/fish, according to the staining intensity of red blood cells in the tail vein of zebrafish (Table 2, Figure 4).

### 2.4. Determination of t-PA Content

**Figure 5:**
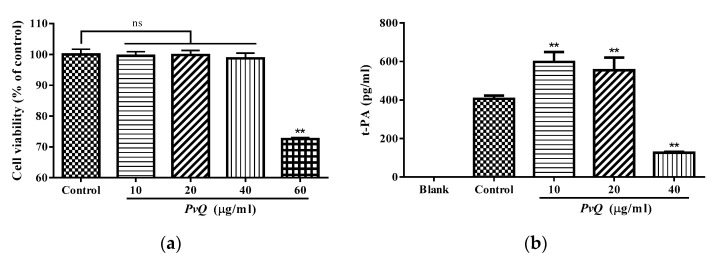
(**a**) CCK-8 assay to evaluate the cell viability of HUVECs treated by *PvQ* for 24 h and (**b**) the expression level of t-PA in the supernatant of HUVECs. Data are expressed as mean ± SD (*n* = 6). ^ns^ *p* > 0.05, ** *p* ≤ 0.01 compared to the control group.

Tissue type plasminogen activator (t-PA) is one of the proteases secreted by vascular endothelial cells which converts the proenzyme plasminogen to plasmin. Increasing the release of t-PA can promote the dissolution of the thrombus. Additionally, t-PA plays an indirect role in thrombolysis and is even given as a drug to relieve ischemia. To verify whether the *PvQ* can promote the release of t-PA as reported in the literature [21], we first conducted a 2-(2-methoxy-4-nitrophenyl)-3-(4-nitrophenyl)-5-(2,4-disulfonic acid benzene)-2H-tetrazolium monosodium salt (CCK-8) assay to examine the cell viability in different treatment groups. Then, an ELISA assay was performed in this research. As displayed in Figure 5a, the viability of HUVECs was not affected when the concentration of *PvQ* was lower than 40 µg/mL. Noticeably, the decrease was distinct with 60 µg/mL of *PvQ*. Therefore, the maximum drug-treated concentration was determined as 40 µg/mL. Additionally, the results of the ELISA assay revealed that the amount of t-PA was significantly upregulated (Figure 5b) at a concentration of *PvQ* not greater than 20 µg/mL. Confusingly, t-PA levels declined as the drug concentration increased. In our view, the reason for this phenomenon may be that *PvQ*, as a basic serine protease, has trypsin-like effects and a high dose of *PvQ* hydrolyzed t-PA [22]. To be precise, a definitive explanation of this phenomenon requires further experimentation later. However, in any case, the level of t-PA did increase in the presence of low concentrations of *PvQ*. Meanwhile, earthworm fibrinolytic proteins were orally administered in the traditional application, and the plasma concentration of the active proteases may not reach a high level, thus, showing a therapeutic effect rather than hydrolysis.

### 2.5. Purification of Functional Proteins from PvQ

**Figure 6:**
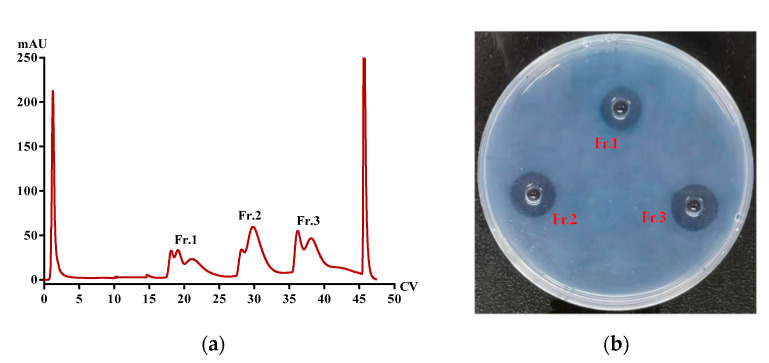
(**a**) HiTrap Q HP-chromatographic pattern. The bound proteins were eluted from 0 to 20 mM NaCl solution. (**b**) The fibrinolytic activity of three fractions.

**Figure 7:**
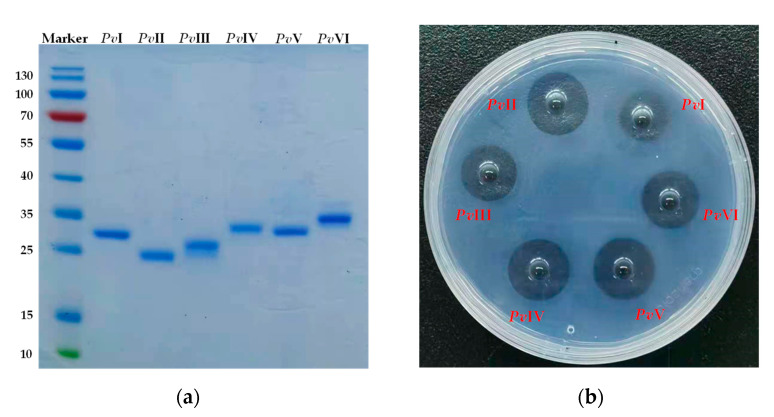
(**a**) Profiling of six novel *P. vulgaris-*derived proteases; and (**b**) the fibrinolytic activity of each protease.

The freeze-dried *PvQ* was redissolved in 20 mM Tris-HCl buffer (pH 7.8) and subjected to a HiTrap Q HP column. Three fractions (Fr.1–Fr.3) possessing fibrinolytic activity were collected (Figure 6) and the SDS-PAGE profile of the fractions showed impurities. Fr.1–Fr.3 were successively loaded into a HiPrep Phenyl FF column. After collecting all chromatographic peak fractions, the fibrin plate assay was performed to verify their activity. The results showed that Fr.1–Fr.3 contained 1, 2 and 3 active peaks (Fr.1a, Fr.2a, Fr.2b, Fr.3a–Fr.3c), respectively. Then, gel filtration chromatography (Superdex 75 Increase) was carried out for all the above active fractions. Finally, six novel *P*. *vulgaris*-derived fibrinolytic proteins, named *Pv*I–*Pv*VI, were obtained from *PvQ*, with the molecular weight ranging from 20 to 35 kDa. Additionally, most bands were more than 98% purity as estimated by SDS-PAGE coomassie brilliant blue staining (Figure 7a). Meanwhile, under approximately the same concentration conditions (0.23 mg/mL, determined by the bicinchoninic acid method), the six proteases possessed significant biological activity, proven by the fibrin plate assay. The rank order of the fibrinolytic activity of the six isoenzymes on the fibrin plates is *Pv*II > *Pv*V > *Pv*IV > *Pv*VI > *Pv*III > *Pv*I (Figure 7b).

### 2.6. Structural Identification of PvI–PvVI

#### 2.6.1. The Bottom-Up Proteomics and N-Terminal Sequence Analysis

**Figure 8:**
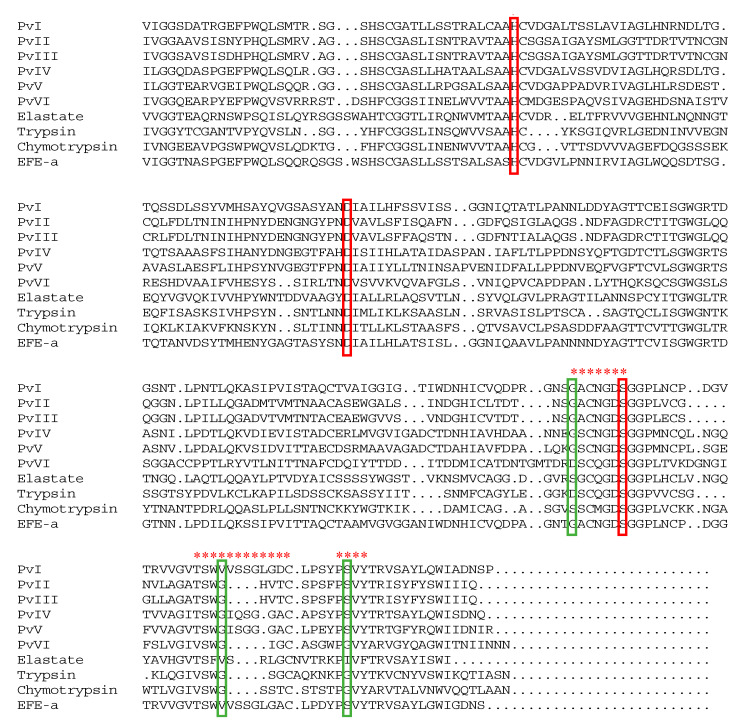
Sequence alignment of six purified proteins, bovine chymotrypsin, bovine trypsin, and porcine elastase. The amino acid residues of the catalytic triad (red box) and the entrance of the substrate-binding pocket (green box) are represented. The residues in the S1 pocket are marked with red asterisks.

Bottom-up proteomic strategy is far more widespread and refers to using exhaustive, usually tryptic, digestion of proteins prefractionated by polyacrylamide gel electrophoresis. In the present study, six trypsin-digested samples were injected into LC-MS/MS and the results were analyzed using Byonic^TM^ compared against the local database of *P*. *vulgaris*. The retrieval results for the six proteins are shown in Table 3 and all proteins have more than two unique peptides. Next, we determined the PTH amino acid compositions which were released in each step of Edman degradation. The N-terminal sequence of the first 10 amino acid residues of purified proteases from *PvQ* are shown in Table 4 and the chromatograms are shown in the Supplementary Materials Figures S1–S8. Consequently, the actual amino acids sequence was identified from the possible sequences based on Byonic^TM^ combined with N-terminal sequencing through the Edman degradation assay. The primary sequences of the mature peptides are shown in the Supplementary Materials Tables S1–S6. Additionally, the six isoenzymes possess sequence similarity of 56.4% and had the same catalytic triplet as trypsin (His^57^, Asp^102^ and Ser^195^), indicating that the functional proteins belong to the trypsin family (Figure 8).

#### 2.6.2. Secondary-Structure Prediction

**Figure 9:**
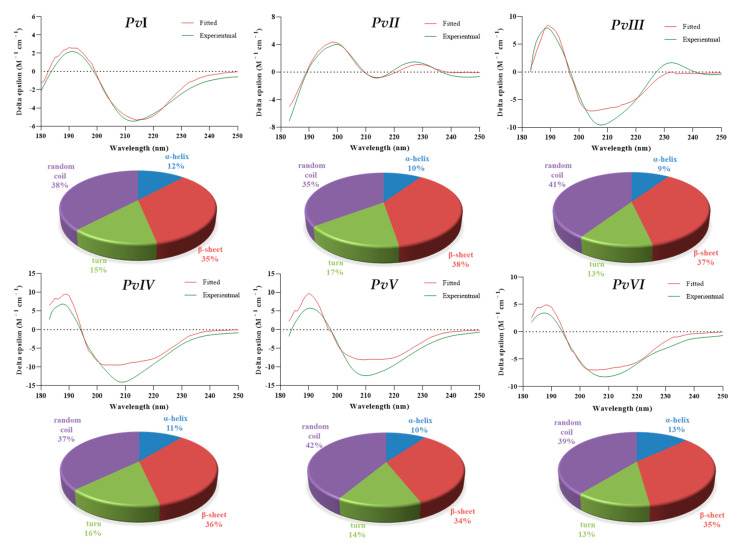
Modelled CD spectra of purified by BeSTSel online tool. The spectra comprise experimental values (green color) and reconstituted values (red color) and the pie charts represent different forms of secondary structure in purified proteases.

Circular Dichroism (CD) spectroscopy is a widely used technique for the study of protein structure. The results through BeSTSel algorithms [23] showed that the secondary structure of the proteins covers various forms, including *α*-helix, *β*-sheet, turn and random coil (Table 5). As the results in Figure 9 show, the secondary structure forms in all six proteases have a similar tendency, of which strands and random coil account for the largest proportion. Additionally, a high proportion of unordered secondary structures in earthworm fibrinolytic protease could be the reason for the broad substrate affinity of the enzyme and stability [13].

#### 2.6.3. Tertiary-Structure Prediction

**Figure 10:**
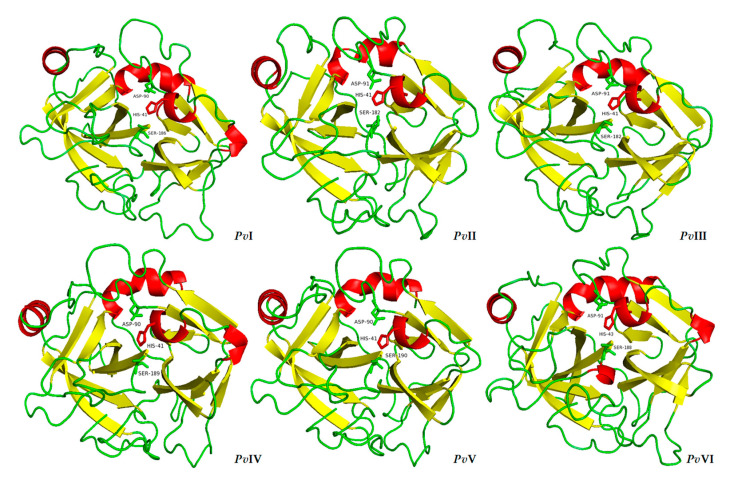
The 3D structures of *Pv*I–*Pv*VI. Each molecule consists of *α*-helix (red column), *β*-sheets (yellow arrows) and unordered regions (green thread) that form two super-secondary structure motifs and one cleft. Active site residues (Ser, His and Asp) are in the cleft as indicated in the figure.

**Figure 11:**
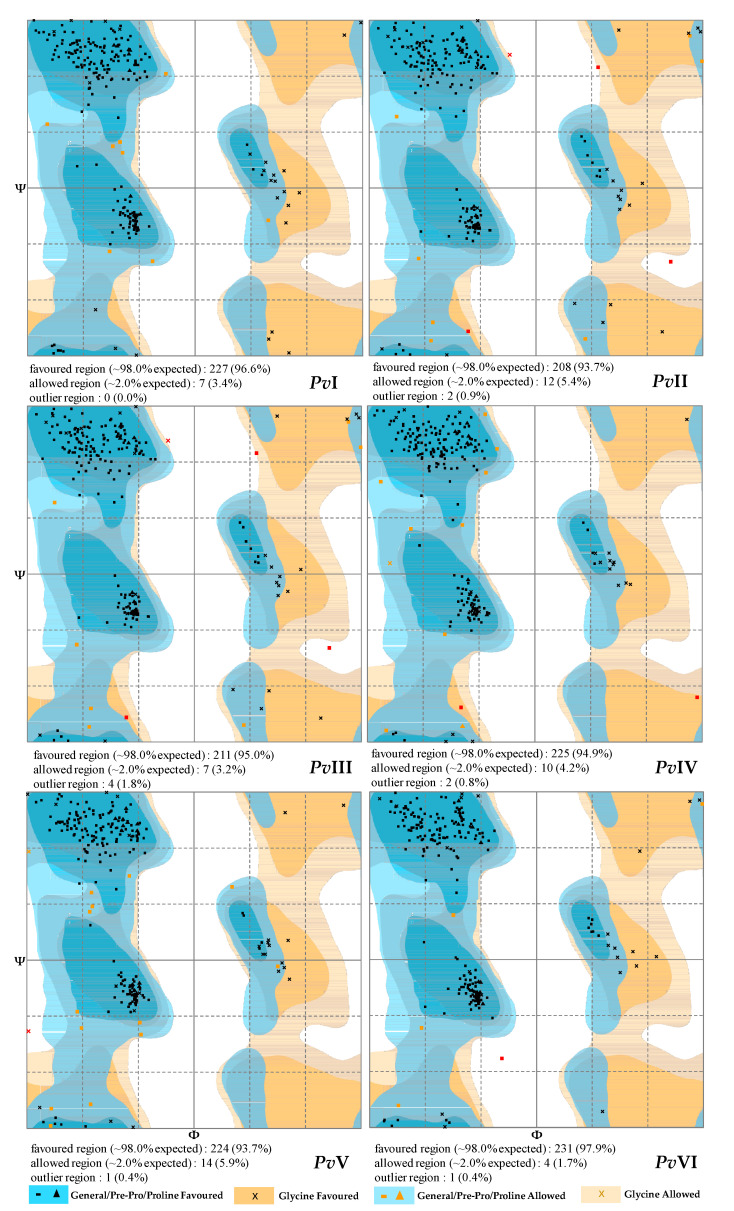
The PROCHECK Ramachandran plot of *Pv*I–*Pv*VI by RAMPAGE online software.

MODELLER 9.23 was used to predict the three-dimensional model of the six proteases. The results of BLASTtp before modelling showed that the purified proteases had a high similarity with the homologous template in the PDB database, ranging fom 36% to 76%. According to the principle of minimum energy, the optimal model was selected from the ten models generated by MODELLER 9.23 and loop refining was performed to obtain the final optimization model (Figure 10). Furthermore, RAMPAGE software verified the validity of the six selected models and a Ramachandran plot of them is shown in Figure 11. The percentage of residues in the most favored regions and in the allowed regions of each protein is above 95%. Therefore, the result of this modelling prediction carries a high level of confidence.

**Figure 12:**
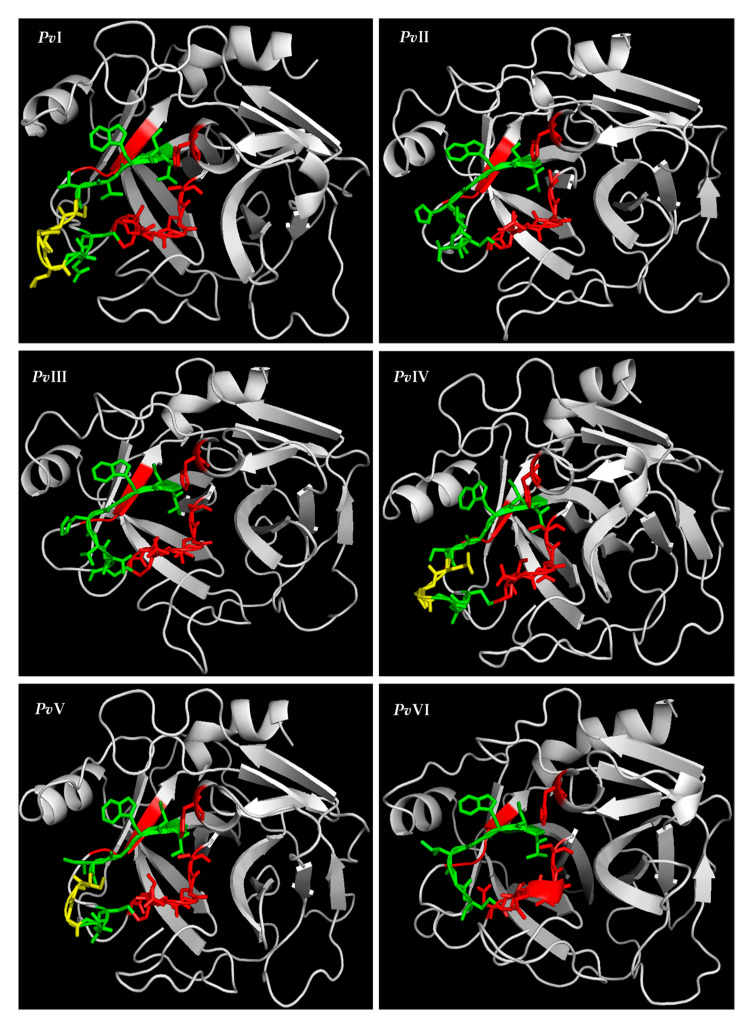
The S1 specificity pocket of six predicted 3D structures are shown in the colored regions.

The resulting homology model showed that each of the six proteases contains two *β*-sheet barrel-like subdomains connected by three trans-domain straps. The overall structure is very similar to that of the trypsin family. As shown in Figure 10, except for *Pv*I, which contains two six-stranded barrels, the N-terminal barrel contains six antiparallel *β*-sheets and the C-terminal barrel contains seven in the *Pv*II–*Pv*VI. The active site cleft and the catalytic residues are located at the junction of both barrels, with the active site cleft perpendicular to the junction. Moreover, the S1 pocket was walled by several residues, indicated in Figure 8 with red asterisks. As the 3D models of *Pv*I–*Pv*VI showed in Figure 12, the histidine residue is located in the top right of the S1 pocket in *Pv*I–*Pv*VI; the Gly (Asp)–Ser segment goes from the bottom to the upper right; Thr (Val)–Cys goes from the top to the bottom. Besides the segment, Pro–Val is in the center and Tyr is closed at the bottom of S1 pocket. According to the predicted structural information, we know that *Pv*I, *Pv*IV and *Pv*V possess the essential S1 specificity determinants characteristic of elastase, but the amino acid residues at the entrance of their pockets are different from elastase, *Pv*I (Val, Ser), *Pv*IV (Gly, Ser) and *Pv*V (Gly, Ser). This might cause a wider space at the entrance of the substrate-binding pocket than the elastase [24]. In addition, the S1 pockets of *Pv*II and *Pv*III were similar to that of chymotrypsin. However, *Pv*VI has a mostly identical substrate-binding pocket with trypsin, and both of them possess Asp residue at the bottom of their pockets. Therefore, *Pv*VI may have excellent binding ability for Lys and Arg residues [25].

### 2.7. Gene Cloning of Fibrinolytic Proteins

**Figure 13:**
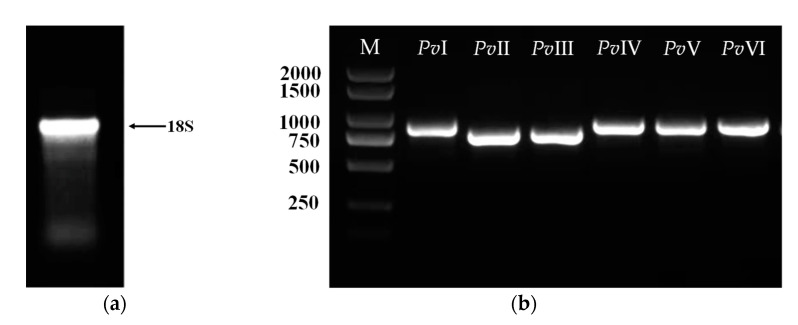
(**a**) The agarose gel electrophoresis of total RNA; and (**b**) six fibrinolytic protein genes of *P. vulgaris*.

**Figure 14:**
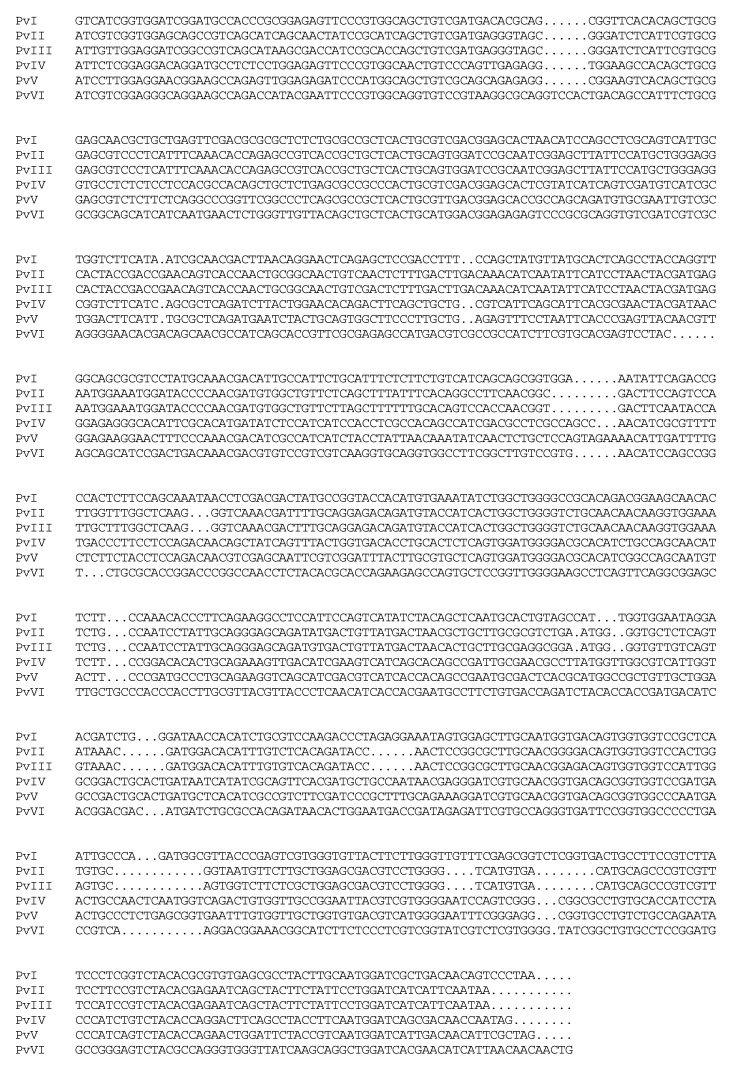
The gene sequences of six proteases, *Pv*I–*Pv*VI, from *P*. *vulgaris*.

Total RNA was obtained through a commercial kit, and agarose gel electrophoresis was used to detect RNA integrity. Figure 13a shows tight 18S ribosomal electrophoretic bands, indicating the presence of good-quality intact RNA; the OD_260/280_ is 1.89. Next, the cDNA was generated by RT-PCR using total RNA as a template. The six genes of mature peptide sequences were cloned in succession by specific primers and cDNA, as shown in Figure 13b. The resultant DNA fragment was inserted into a pCE2 TA/Blunt-Zero vector and the sequencing results of the six genes were illustrated in Figure 14. The open reading frame (ORF) sequence of *Pv*I–*Pv*VI contains 238, 225, 225, 240, 242, and 239 codons including a stop codon, respectively.

Additionally, the full-length sequences of these six genes were cloned based on transcriptome data in previous studies. However, based on the N-terminal amino acid sequence, we found the proteases might consist of duplicate regions, including a mature peptide sequence and a pro-region sequence upstream from its mature sequence, as previously reported [26,27]. The active form (the mature protein) of *Pv*I–*Pv*VI is initiated from isoleucine or valine but not methionine, which implies that the polypeptides produced may be conducted through post-translational modification. Meanwhile, the primary sequence of proteases was further verified by cloning the genes. Additionally, these six fibrinolytic genes (Supplementary Materials Tables S7–S12) were reported for the first time, providing a research basis for the subsequent heterologous expression of such fibrinolytic proteins derived from earthworm.

## 3. Discussion

Although many fibrinolytic agents have been developed and used for clinical purposes, thromboembolic diseases remain the leading cause of adult morbidity and mortality in the world [3]. Increasing attention has been applied in recent years to the development of effective agents for clinical application from various unusual animal species [2]. A group of fibrinolytic enzymes secreted by the alimentary tract of earthworm have exhibited excellent potential in the clinical treatment of blood clotting diseases [28]. However, the study of medicinal animals has always been a knotty problem in traditional medicine, which has led to insufficient research on their bioactive constituents and clinical application.

In this research, we established a time-saving purification process for the enrichment of active extracts from *P. vulgaris*, a medicinal animal listed in the Chinese pharmacopoeia, and denominated as *PvQ*. In the in vitro study, the fibrin plate assay was performed to verify its fibrinolytic activity. During fibrin–agarose coagulation, thrombin converts fibrinogen to fibrin monomers, which connect to form fibrin bundles. This process is very similar to that of blood coagulation [29]. The area of clear hydrolyzed zone on the fibrin plate indicated that *PvQ* had greater bioactivity than lumbrokinase and this activity difference may result from the varieties of earthworm species. Additionally, *PvQ* exhibited an excellent in vivo thrombolysis effect through the thrombotic zebrafish model. Unfortunately, restricted by low yield, antithrombotic and thrombolytic effects in mammalian models have not yet been evaluated. Furthermore, the proteases have the ability not only to hydrolyze fibrin but also to activate proenzymes, such as plasminogen [21,30]. In the present study, the ELISA assay indicated that *PvQ* can promote the secretion of t-PA in HUVECs. This result agreed with the traditional application of earthworm protein extract for the prevention of thrombotic diseases. Certainly, given the whole complexity of endothelial cells, more cell model experiments and in vivo experiments are needed to demonstrate the potential activity of *PvQ*.

It is evident from various studies that lumbrokinase derived from earthworm has potential proteolytic activity and has been used to cure cardiovascular diseases [31] since ancient times. However, research on the material basis of its efficacy is relatively insufficient, and the identification of protein structures remains a major challenge. Therefore, the present study was conducted to isolate and identify earthworm fibrinolytic enzymes derived from *P. vulgaris* to be used for the treatment of thrombotic diseases. Fortunately, six novel *P*. *vulgaris*-derived fibrinolytic proteases were obtained from the active extract, *PvQ*, in the current research. Their primary sequences were identified through LC-MS/MS transcriptome cross identification and the Edman degradation assay. Analysis of conserved sequence motifs showed that the six proteases had the same catalytic triplet as the trypsin family. Meanwhile, the circular dichroism spectra and homologous modelling were performed to predict the spatial structures. According to the predicted structural information, we know that *Pv*I, *Pv*IV and *Pv*V possess typical S1-pocket of elastase-like protease, which suggests that their S1 pockets should be preferable only for P1 residues with small and hydrophobic side-chains. In addition, the S1 pockets of *Pv*II and *Pv*III were similar to those of chymotrypsin. However, compared to the substrate-binding pocket structures of *Pv*II and *Pv*III with chymotrypsin, Gly^216^ and Gly^226^ at the entrance of the substrate-binding pocket of chymotrypsin are replaced by Gly and Ser in *Pv*II and *Pv*III. These extra side chains in *Pv*II and *Pv*III make the entrance narrower than that of chymotrypsin. The backbones of the S1 pocket in *Pv*VI are readily superimposable on that of trypsin and the Asp residues at the bottom of the pockets improved the substrate specificity of *Pv*VI. In short, structural research is significant for comprehending protein functions, biological mechanisms and the interaction of proteins, which is very important for future exploration in biological medicine and pharmaceutics. Therefore, it will be an important project to culture protein crystals in the following studies.

Although the monomers can be separated by conventional purification methods, it is a time-consuming and difficult project. The use of different purification procedures will also result in a final product that varies in composition. Therefore, the level of fibrinolytic activity may also complex. Therefore, the successful expression of the recombinant protease provides a new approach to obtain a single component with fibrinolytic activity [32,33,34]. To date, 42 earthworm fibrinolytic gene sequences are publicly available at NCBI GenBank, including *Eisenia foetida* [35], *Lumbricus rubellus* [36], among others. A few of these genes have also been successfully expressed and characterized in *E. coli* [35,36,37] and the yeast *Pichia pastoris* [33,38,39]. Certainly, the majority of studies have reported that, for undetermined reasons, recombinant proteins are either not expressed or do not exhibit fibrinolytic activity. Additional studies are required to clarify why some genes are capable of being expressed in cell culture systems, but without fibrinolytic activity. In the current study, six fibrinolytic genes encoding fibrinolytic proteins derived from *P*. *vulgaris* have been reported for the first time. Additionally, the *Pichia pastoris* expression system is being used to explore the expression of target proteins, but the results remain unpredictable.

Although earthworm fibrinolytic proteases have some advantages in pharmaceutical applications, there are some problems that need to be solved. First, as an oral drug, it is not clear how earthworm fibrinolytic protease passes through the intestinal mucosa and transport into the blood circulation. Meanwhile, it is ambiguous whether earthworm fibrinolytic proteases will produce plasmin complications when they directly dissolve fibrinogen and fibrin. Additionally, the molecular mechanism for its thrombolytic effect in vivo remains unclear. Therefore, it is necessary to generate the fibrinolytic monomer and study its pharmacokinetics and toxicology in future research. It is well-known that the molecular structure is the basis of its catalytic mechanism and determines their extraordinary substrate specificity, so the study of bioactive protein spatial structure contributes to a better understanding of the fibrinolysis mechanisms. In conclusion, biophysical studies are necessary for the elucidation of their molecular biological characteristics and therapeutic applications.

## 4. Materials and Methods

### 4.1. Material

Fresh *P. vulgaris* was collected from Shanghai, China. A voucher specimen, code Pv-Dsy 2020, was deposited at the School of Chinese Materia Medica, Beijing University of Chinese Medicine. Additionally, their crude extract was obtained according to laboratory preparation technology. Lumbrokinase (140650–201804), fibrinogen (140607–202042), thrombin (140605–201927) were all acquired from the Chinese Food and Drug Testing Institute. High-glucose Dulbecco’s modified Eagle medium (DMEM), fetal bovine serum (FBS), penicillin and streptomycin were purchased from Gibco (Thermo Fisher Scientific Co., Waltham, MA, USA). A human t-plasminogen activator ELISA kit (KE00180) was purchased from Wuhansanying Biotechnology Co. Ltd. (Proteintech, Wuhan, China). Other applied reagents were of analytical grade or above.

### 4.2. Enrichment of the Fibrinolytic Extract, PvQ

The lyophilized-crude extract powders (200 mg) were dissolved in 50 mL of a 20 mM Tris-HCl buffer (pH 7.8), filtered with membrane filters (0.45 µm) and loaded on a prepacked HiTrap Q HP column (GE Healthcare, Chicago, IL, USA) equilibrated with the buffer. After loading, the adsorbed proteins were eluted with a stepwise gradient of 0.1–1 M NaCl in the Tris-HCl buffer at a flow rate of 5.0 mL per minute on the AKTA pure. At the end of elution, all fractions were collected, and a Fibg-TT assay was carried out to evaluate their bioactivity. According to the results of the Fibg-TT assay, the anticoagulant fractions were screened and desalted via centrifugal filters (MWCO of 3 kD, Millipore, Germany). To facilitate the subsequent experiments, all target fractions were freeze-dried.

### 4.3. Fibrin Plate Assay

The fibrin-hydrolysis activity of *PvQ* was measured by the fibrin plate assay [40,41], with slight modification of the amount of sample. Fibrinogen solution (2 mg/mL) was prepared in 8 mL of normal saline, then mixed with 20 mL of 0.5% agarose solution. Additionally, 200 μL of 40 BP/mL thrombin solution was distributed into each sterile Petri dish before 28 mL of fibrinogen and agarose mixed solution were added. After the agarose solution solidified, samples and serial concentrations of lumbrokinase (24,000, 16,000, 12,000, 8000, 6000, 4000 U/mL) were added to the dish, respectively. The plates were then incubated at 37 °C for 18 h. Two diameters on the X and Y directions of each hydrolyzed clear zone were measured and the hydrolytic activity of *PvQ* was then calculated.

### 4.4. The Effect of PvQ on Zebrafish Thrombotic Model of Vascular Damage

Wildtype (AB) zebrafish were purchased from the China Zebrafish Resource Center, and bred in the zebrafish circulating aquaculture system (ESEN, China) according to Mustafa et al. [42]. Larval zebrafish (5 days post-fertilization) with normal development were selected under a stereomicroscope and randomly placed in 6-well plates containing 3 mL of culture medium, with 10 fishes in each well. The zebrafish in the blank group were cultured normally, and the other groups were treated with epinephrine hydrochloride solution with the final concentration of 30 μM for 16 h in the dark. At the end of the experiment, the fibrinolytic effect of *PvQ* (10 ng/fish) on zebrafish was evaluated by o-anisidine staining; tail vein thrombosis in zebrafish and the reduction in intensity of erythrocyte staining were taken as indicators.

According to the pre-experiment results, the whole laboratory was divided into the model group, the drug administration group and the blank group, and all samples were placed in a 28.5 °C constant temperature biochemical incubator. The experiment was repeated three times with larval zebrafish from different parents. After staining, zebrafish in each group were photographed via a stereoscopic microscope, and the staining intensity of red blood cells in the tail of zebrafish was counted using Image-Pro Plus 6 processing software. The inhibition ratio was expressed through the rate of staining intensity of the *PvQ* group and that of the model group; before calculations, both groups’ background values were subtracted [43].

### 4.5. The Determination of t-PA Content

The human umbilical vein endothelial cell line (No. 1101HUM-PUMC000437) was obtained from the Cell Resource Center of Peking Union Medical College (Beijing, China). HUVECs were cultured in DMEM basal medium supplemented with 10% fetal bovine serum (FBS), 100 IU/mL of penicillin, and 100 µg/mL of streptomycin. The cells were maintained in standard conditions (37 °C, 5% CO_2_, saturated humidity) and the culture medium was renewed every three days. Moreover, subculture was performed at a ratio of 1:3 when the confluence of cells reached about 90%.

The CCK-8 assay kit (Vazyme, Nanjing, China) was used to determine cell viability. HUVECs were seeded in 96-well microplates (Corning, NY, USA) at a density of 10^5^ cells/mL with a total volume of 100 µL for 24 h. The seeded cells were then treated with *PvQ* in different concentrations (ranging from 10 µg/mL to 60 µg/mL, dissolved in DMEM) and incubated for 24 h. Subsequently, 10 μL of CCK-8 solution was added into each well and incubated at 37 °C for another 2 h in the dark. The OD values were measured with a microplate reader (BioTek, Winooski, VT, USA) at 450 nm and cell viability was calculated. On this basis, the maximum drug-treated concentration was determined as cell viability greater than 95% during 24 h of incubation. Under the same conditions, the concentration of t-PA in the cell culture supernatants of each group was assessed using a human t-Plasminogen activator ELISA kit according to the manufacturer’s instructions.

### 4.6. Purification of Fibrinolytic Proteases (PvI–PvVI) from PvQ

*PvQ* is a significant fibrinolytic enrichment, containing several active protein monomers. In order to clarify the effective substances of *PvQ* more thoroughly, a systematic study of its proteases in the fraction was performed in this project and a series of chromatographic techniques were used for purification [12].

Firstly, the lyophilized powders of *PvQ* were redissolved in 20 mM Tris-HCl buffer (pH 7.8) and purified on a HiTrap Q HP column (GE Healthcare, Chicago, IL, USA) eluted with a NaCl gradient from 0 to 20 mM at a flow rate of 5.0 mL/min. The fractions were pooled and assayed by the fibrin plate to track the active protein peak. The eluted active fractions were successively subjected to a HiPrep Phenyl FF column (GE Healthcare, Chicago, IL, USA), eluting with a stepwise gradient of 20 mM Tris-HCl buffer with 1 M ammonium sulfate (from 1 to 0.5 M, pH 7.8). Subsequently, size exclusion chromatography using a Superdex 75 Increase gel filtration column (GE Healthcare, Chicago, IL, USA) was carried out for each active HIC fraction through an AKTA pure protein purification system with 20 mM Tris-HCl buffer containing 20 mM NaCl at a flow rate of 0.5 mL/min. All columns were used repeatedly until SDS-PAGE showed single bands. Active fractions were pooled, desalted, and lyophilized.

### 4.7. Identification of Functional Proteins, PvI–PvVI

#### 4.7.1. Proteins Sequencing Using LC-MS/MS

Six protein monomers, named *Pv*I*–Pv*VI, were identified by bottom-up proteomics using LC-MS/MS and the acquired data were compared with the local database constructed through the transcriptome results of *P*. *vulgaris*. Briefly, six samples were subjected to a 12% polyacrylamide gel and corresponding strips were collected. Six bands were digested in PAGE by trypsin and separated using Ultimate 3000 RSLC (Thermo, Waltham, MA, USA) with the Acclaim PepMap C18 column (1.9 μm, 100 Å, 150 μm i.d. × 150 mm). The loaded sample was gradient eluted from buffer A (0.1% formic acid in water) to buffer B (0.1% formic acid in acetonitrile) at 600 nL/min for a total of 66 min and analyzed by a Q Exactive^TM^ hybrid Quadrupole Orbitrap^TM^ mass spectrometer with spray voltage of 2.2 kV, capillary temperature of 320 °C. Furthermore, Byonic [44] was used to compare the original mass spectrometric data with the local database of *P*. *vulgaris*. Afterwards, the raw data collected by mass spectrometry was retrieved from the local database by Byonic, and results for protein identification were obtained. The nucleotide sequences corresponding to the proteins were also acquired.

#### 4.7.2. N-Terminal Sequence Analysis

**Figure 15:**
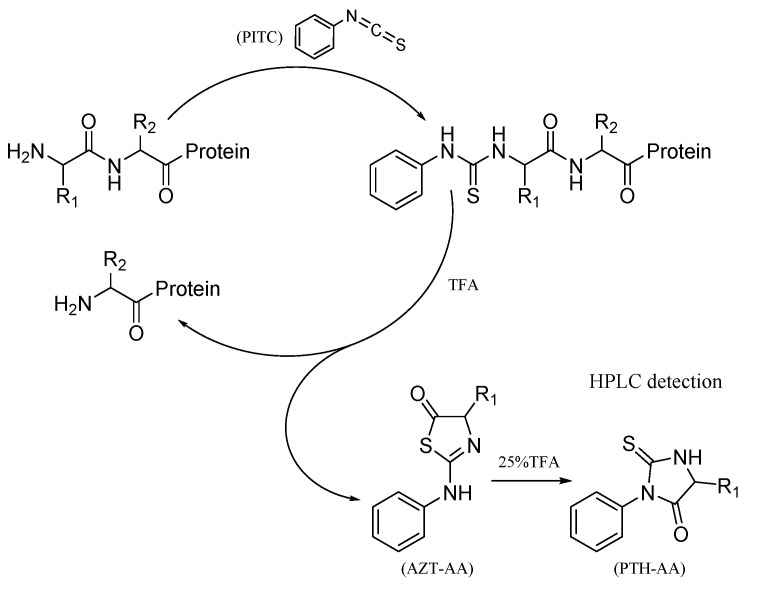
N-terminal sequencing cycle in the Edman degradation assay.

In this study, the Edman degradation assay was used to determine the natural N-terminal sequence of those six proteins. The experimental procedure is presented in Figure 15. More specifically, the protein bands on the SDS-PAGE gel were electroblotted onto a polyvinylidene fluoride (PVDF) membrane. The protein on the film reacted with phenyl isothiocyanate (PITC) using the PPSQ-33A automatic protein sequencer (Shimadzu, Kyoto, Japan) to produce phenylthiohydantoin amino acid (PTH-AA). Afterwards, the PTH-AA was separated via HPLC and identified through comparative analysis with the chromatogram of 19 mixed PTH-AA. The remaining protein was repeatedly treated using the same method as before to produce various PTH-AA in turn. In this experiment, the number of cycles was set to 10 times and raw data were analyzed by the software PPSQ-30 Data Processing.

#### 4.7.3. Secondary Structure Determination

The secondary structure of the six purified proteins was determined by circular dichroism (CD) spectroscopy. CD spectra were carried with about 0.2 mg/mL of purified proteins using a 1.0 mm quartz cell. A CD spectrometer (JASCO 715, Tokyo, Japan) was set with a measurement range of 180*–*250 nm at a scanning speed of 50 nm/min. The bandwidth was 1.0 nm, and the data pitch was 0.1 nm. Deionized water was used as a solvent and to mark the baseline. Further analysis of the results was carried out using BeSTSel, a non-commercial web server (http://bestsel.elte.hu) (accessed on 14 April 2021). This method even estimates *α*-helix content more accurately than previous methods, but it provides detailed information regarding the *β*-sheets, and overcomes the challenge brought about by the large spectral and structural diversity of *β*-sheets [23,45].

#### 4.7.4. Three-Dimensional Protein Structure Prediction

Steric structures of proteins are important for analyzing protein function. Builiding protein crystals has long been a major challenge, and homologous modelling is a quick and reliable method for predicting protein structure when X-ray crystallography or nuclear magnetic resonance (NMR) is not feasible. In this experiment, we predicted the tertiary structures of six novel purified fibrinolytic proteins based on MODDLER 9.23 for multi-template modelling. Firstly, protein homology searches were performed in the Protein Data Bank (PDB) database using a Blastp tool to obtain homologous templates. Subsequently, multi-template modelling was carried out based on the alignment results and structural information of homologous proteins. Then, the optimal model was selected by DOPE scoring in combination with Molpdf score, and loop correction was performed to pick a model with the lowest energy. Furthermore, the RAMPAGE server (http://www-cryst.bioc.cam.ac.uk/rampage) (accessed on 16 March 2021) was used for the validation of the predicted structures [46,47].

### 4.8. Cloning of the Six Genes Proteins Gene

Total RNA was extracted from a living earthworm using the Mollusc RNA kit R6875 purchased from OMEGA Bio-tec, America. Then, reverse transcriptase-polymerase chain reaction (RT-PCR) was conducted according to the protocol of the HiScript III 1st Strand cDNA Synthesis kit (Vazyme, Nanjing, China) with the oligo (dT)_20_ primer. Upon the nucleotide sequence of the transcriptome of *P. vulgaris*, six pairs of primers (Table 6) were designed to be used in PCR amplification with cDNA by the Phanta Max Super-Fidelity DNA Polymerase kit (Vazyme, Nanjing, China). PCR was performed with 3 µL cDNA template, 1 µL DNA polymerase, 1 µL dNTP mix, and 2 µL of each primer in a 50 µL reaction mixture. Additionally, the PCR protocol was as follows: a pre-denaturation for 3 min at 95 °C was followed by 35 cycles of 15 s at 95 °C, 15 s at the right temperature (*Tm*), and 1 min at 72 °C. The PCR product was purified by the Gel/PCR extraction kit (Biomiga, San Diego, CA, USA) and subcloned into a pCE2 TA/Blunt-Zero vector at 37 °C using the 5 min TA/Blunt-Zero Cloning kit (Vazyme, Nanjing, China). The recombinant plasmids were transformed into *E. coli* DH5*α* and sequenced. The sequencing results were compared with the sequences in the transcriptome to further verify the validity of the primary sequences of the purified proteins.

### 4.9. Statistical Analysis

All statistical analyses were performed through one-way ANOVA with the aid of SPSS 20.0; results were presented as mean ± SD. A *p*-value under 0.05 was regarded as a significant difference.

## 5. Conclusions

In this paper, a bioactive protein fraction, *Pv*Q, was isolated from *P. vulgaris* using AKTA pure, which exhibited excellent in vitro and in vivo activity. In order to clarify the effective substances of *PvQ* more thoroughly, a systematic study of its fibrinolysis constituents was performed, combined with extensive column chromatography techniques. Until now, six novel bioactive proteases were obtained and their primary structures were identified through mass spectrometry techniques in combination with RNA-seq. Moreover, the spatial structures were developed by CD spectroscopy and homology modelling methods. Six genes encoding purified proteases were cloned in succession, which laid the foundation for subsequent heterologous expression and enriched the fibrinolytic gene data from *P. vulgaris*.

## Figures and Tables

**Table 1:** The specific activity of *PvQ* (Mean ± SD, *n* = 3).

No.	Specific Activity (U/μg) ^1^		
	*PvQ*-1	*PvQ-*2	*PvQ-*3
1	243.01	246.33	250.15
2	257.36	265.26	248.62
3	250.78	230.82	233.65
Mean ± SD	247.33 ± 10.74		

^1^ Specific activity indicated the ratio of activity concentration (U) versus the soluble protein concentration (C).

**Table 2:** The staining intensity of tail vein erythrocytes (Mean ± SD, *n* = 18).

Group	Breeding Conditions	Staining Intensity
Normal	Culture medium	2064.96 ± 152.12
Model	Adrenaline hydrochloride solution (30 μM)	2412.01 ± 131.94 **
*PvQ*	*PvQ* 10 ng/fish	2178.33 ± 186.91 *^##^

* *p* ≤ 0.05, ** *p* ≤ 0.01 compared to the normal group; ^##^ *p* ≤ 0.01 compared to the model group.

**Table 3:** The identified proteins compared against the local database of *P. vulgaris*.

Sample	Coverage (%)	^#^ of Spectra	Unique Peptides	Score
*Pv*I	26.87	470	6	81.46
*Pv*II	24.84	507	7	90.87
*Pv*III	17.93	385	5	56.43
*Pv*IV	8.78	53	3	19.73
*Pv*V	34.12	407	10	70.53
*Pv*VI	25.22	82	9	34.21

^#^ Number of identified peptides of this protein.

**Table 4:** N-terminal amino acid sequences of *Pv*I–*Pv*VI.

Sample	N-terminal Amino Acid Sequences
*Pv*I	Val-Ile-Gly-Gly-Ser-Asp-Ala-Thr-Arg-Gly
*Pv*II	Ile-Val-Gly-Gly-Ala-Ala-Val-Ser-Ile-Ser
*Pv*III	Ile-Val-Gly-Gly-Ser-Ala-Val-Ser-Ile-Ser
*Pv*IV	Ile-Leu-Gly-Gly-Gln-Asp-Ala-Ser-Pro-Gly
*Pv*V	Ile-Leu-Gly-Gly-Thr-Glu-Ala-Arg-Val-Gly
*Pv*VI	Ile-Val-Gly-Gly-Gln-Glu-Ala-Arg-Pro-Tyr

**Table 5:** The result of predicted secondary structure in purified protease.

Sample	*α*-Helix (%)	*β*-Sheet (%)	Turn (%)	Random Coil (%)
*Pv*I	11.8	35.0	15.2	38.0
*Pv*II	9.4	38.0	17.4	35.2
*Pv*III	9.4	37.0	12.9	40.7
*Pv*IV	10.9	35.6	16.3	37.2
*Pv*V	10.0	34.0	14.5	41.5
*Pv*VI	13.0	34.8	13.0	39.2

**Table 6:** Six pairs of specific primer sequences.

Protein	Primer	Primer Sequence	*Tm* (°C)
*Pv*I	*Pv*I-F *Pv*I-R	5′-GTCATCGGTGGATCGGATGC-3′ 5′-TTAGGGACTGTTGTCAGCGATCC-3′	55.9 57.1
*Pv*II	*Pv*II-F *Pv*II-R	5′-ATCGTCGGTGGAGCAGCCGTCAG-3′ 5′-TTATTGAATGATGATCCAGG-3′	62.4 45.6
*Pv*III	*Pv*III-F *Pv*III-R	5′-ATTGTTGGAGGATCGGCCGTCAG-3′ 5′-TTATTGAATGATGATCCAGG-3′	58.8 45.6
*Pv*IV	*Pv*IV-F *Pv*IV-R	5′-ATTCTCGGAGGACAGGATGCCTC-3′ 5′-CTATTGGTTGTCGCTGATCCAT-3′	58.8 53.0
*Pv*V	*Pv*V-F *Pv*V-R	5′-ATCCTTGGAGGAACGGAAGCCAG-3′ 5′-CTAGCGAATGTTGTCAATGATCC-3′	58.8 53.5
*Pv*VI	*Pv*VI-F *Pv*VI-R	5′-ATCGTCGGAGGGCAGGAAGC-3′ 5′-TCAGTTGTTGTTAATGATGTTCG-3′	57.9 50.0

## Data Availability

The study did not report any data.

## Supplementary Materials

The following are available online, Figure S1: Chromatography of 19 phenylthiohydantoin amino acid mixtures for PvI, Figure S2: Chromatography of N-terminal amino acids of PvI at positions 1–5 (a), 6–10 (b), Figure S3: Chromatography of 19 phenylthiohydantoin amino acid mixtures for PvII-PvVI, Figures S4–S8: Chromatography of N-terminal amino acids of PvII–PvVI at positions 1–5 (a), 6–10 (b), Tables S1–S6: The amino acids sequence of PvI–PvVI, Tables S7–S12: The gene sequences of *Pv*I–*Pv*VI.

## Sample Availability

Samples of the compounds *Pv*I–*Pv*VI are available from the authors.

## References

[1] Weitz J.I., Eikelboom J.W. (2016). Advances in thrombosis and hemostasis. Circ. Res..

[2] Zhao H., Chen J., Jin F., Wu X., Zhu Z., Zhang J., Zhao Y. (2015). Animal drugs in treatment of cerebral ischemia and their mechanisms. Int. J. Pharma Sci. Res..

[3] Wu Y., Hu S., Ma Y., Zhao B., Yang W., Lu Y., Li P., Du S. (2020). Novel *Pheretima guillelmi*-derived antithrombotic protein DPf3: Identification, characterization, in vitro evaluation and antithrombotic mechanisms investigation. Int. J. Biol. Macromol..

[4] Marc V. (2000). Third-generation thrombolytic drugs. Am. J. Med..

[5] Wang Y.H., Li S.A., Huang C.H., Su H.H., Chen Y.H., Chang J.T., Huang S.S. (2018). Sirt1 activation by post-ischemic treatment with lumbrokinase protects against myocardial ischemia-reperfusion injury. Front. Pharmacol..

[6] Wang X.M., Fan S.C., Chen Y., Ma X.F., He R.Q. (2019). Earthworm protease in anti-thrombosis and anti-fibrosis. Biochim. Biophys. Acta (BBA)-Gen. Subj..

[7] Yang S.J., Ru B.G. (1997). Purification and characterization of an SDS-activated fibrinolytic enzyme from *Eisenia fetida*. Comp. Biochem. Physiol. Part B.

[8] Matausic-Pisl M., Tomicic M., Micek V., Grdisa M. (2011). Influences of earthworm extract G-90 on haematological and haemostatic parameters in Wistar rats. Eur. Rev. Med. Pharmcol..

[9] Joyia F.A., Zia M.A., Mustafa G., Faheem A., Raana H.T., Khan M.S. (2018). Isolation, purification and functional characterization of fibrinolytic protease from an earthworm *Eisenia foetida*. Pure Appl. Biol..

[10] Cho I.H., Choi E.S., Lim H.G., Lee H.H. (2004). Purification and characterization of six fibrinolvtic serine-proteases from earthworm *Lumbricus rubellu*s. J. Biochem. Mol. Biol..

[11] Trisina J., Sunardi F., Suhartono M.T., Tjandrawinata R.R. (2011). DLBS1033, A protein extract from *Lumbricus rubellus*, possesses antithrombotic and thrombolytic activities. J. Biomed. Biotechnol..

[12] Phan T., Ta T., Nguyen D., Broek L., Duong G. (2011). Purification and characterization of novel fibrinolytic proteases as potential antithrombotic agents from earthworm *Perionyx excavatus*. AMB Express.

[13] Tang M., Liu C., Liang Z., Gong M., Hu D. (2016). Studies on separation and properties of lumbrokinase in *Pheretima praepinguis*. J. Bangladesh. Pharmacol..

[14] Verma M.K., Pulicherla K.K. (2017). Broad substrate affinity and catalytic diversity of fibrinolytic enzyme from *Pheretima posthumous*-purification and molecular characterization study. Int. J. Biol. Macromol..

[15] Mihara H., Sumi H., Yoneta T., Mizumoto H., Ikeda R., Seiki M., Maruyama M. (1991). A novel fibrinolytic enzyme extracted from the earthworm, *Lumbricus rubellus*. Jpn. J. Physiol..

[16] Tjandrawinata R.R., Trisina J., Rahayu P., Prasetya L.A., Hanafiah A., Rachmawati H. (2014). Bioactive protein fraction DLBS1033 containing lumbrokinase isolated from *Lumbricus rubellus*: Ex vivo, in vivo, and pharmaceutic studies. Drug Des. Dev. Ther..

[17] Wu Y., Ma Y., Hu S., Zhao B., Yang W., Sun Z., Zhu B., Lu Y., Li P., Du S. (2019). Transcriptomic-proteomics-anticoagulant bioactivity integrated study of *Pheretima guillemi*. J. Ethnopharmacol..

[18] State Food and Drug Administration (2010). National Drug Standard.

[19] Fan J.J., Li F.R., Zhao C.J., Xu K.X., Yang R., Qiao Y.H., Ni Y.Y., Wang Y.T., Ma Z.Q., Lin R.C. (2017). Optimization and adaptability of the zebrafish thrombosis model induced by adrenaline hydrochloride. Glob. Tradit. Chin. Med..

[20] Fan J.J., Qiao Y.H., Zhao C.J., Ni Y.Y., Yang R., Feng Y.R., Ma Z.Q., Lin R.C. (2017). Applicability of zebrafish thrombosis model in antithrombotic activity screening of Chinese materia medica. Chin. J. Inf. TCM.

[21] Zhao J., Pan R., He J., Liu Y., Li D.F., He R.Q. (2007). *Eisenia fetida* protease-III-1 functions in both fibrinolysis and fibrogenesis. J. Biomed. Biotechnol..

[22] Nakajima N., Sugimoto M., Ishihara K. (2003). Earthworm-serine protease: Characterization, molecular cloning, and application of the catalytic functions. J. Mol. Catal. B-Enzym..

[23] Micsonai A., Wien F., Kernya L., Lee Y.H., Goto Y., Refregiers M., Kardos J. (2015). Accurate secondary structure prediction and fold recognition for circular dichroism spectroscopy. Proc. Natl. Acad. Sci. USA.

[24] Tang Y., Liang D., Jiang T., Zhang J., Gui L., Chang W. (2002). Crystal structure of earthworm fibrinolytic enzyme component A: Revealing the structural determinants of its dual fibrinolytic activity. J. Mol. Biol..

[25] Sugimoto M., Ishihara K., Nakajima N. (2003). Structure and function of an isozyme of earthworm proteases as a new biocatalyst. J. Mol. Catal. B-Enzym..

[26] Cho H., Choi E., Lee H.H. (2004). Molecular cloning, sequencing, and expression of a fibrinolytic serine-protease gene from the earthworm *Lumbricus rubellus*. J. Biochem. Mol. Biol..

[27] Ge T., Sun Z.J., Fu S.H., Liang G.D. (2005). Cloning of thrombolytic enzyme (lumbrokinase) from earthworm and its expression in the yeast *Pichia pastoris*. Protein Expr. Purif..

[28] Zhao J., Xiao R., He J., Pan R., Fan R., Wu C., Liu X., Liu Y., He R.Q. (2007). In situ localization and substrate specificity of earthworm protease-II and protease-III-1 from *Eisenia fetida*. Int. J. Biol. Macromol..

[29] Pieters M., Wolberg A.S. (2019). Fibrinogen and fibrin: An illustrated review. Res. Pract. Thromb. Haemost..

[30] Jin L., Jin H., Zhang G., Xu G. (2000). Changes in coagulation and tissue plasminogen activator after the treatment of cerebral infarction with lumbrokinase. Clin. Hemorheol. Microcirc..

[31] Grdisa M., Mikecin A., Hrzenjak T. (2009). Earthworms as a source of bioactive molecules. Curr. Bioact. Compd..

[32] Zhao G., Li H., Xu W., Luo J., Xu R.A. (2010). An overview of the fibrinolytic enzyme from earthworm. Chin. J. Nat. Med..

[33] Yuan X., Cao C., Shan Y., Zhao Z., Chen J. (2006). Expression and characterization of earthworm *Eisenia foetida* Lumbrokinase-3 in *Pichia pastoris*. Prep. Biochem. Biotechnol..

[34] Ueda M., Hirano Y., Fukuhara H., Naka Y., Nakazawa M., Sakamoto T., Ogata Y., Tamada T. (2018). Gene cloning, expression, and X-ray crystallographic analysis of a *β*-mannanase from *Eisenia fetida*. Enzym. Microb. Technol..

[35] Li G.Q., Wang K.Y., Li D.H., Wang N., Liu D.H., Jitesh P. (2012). Cloning, expression and characterization of a gene from earthworm *Eisenia fetida* encoding a blood-clot dissolving protein. PLoS ONE.

[36] Li D., Tong W., Yang Y. (2008). Functional expression of an earthworm fibrinolytic enzyme in *Escherichia coli*. World J. Microb. Biotechnol..

[37] Xu Z., Yang Y., Gui Q., Zhang L., Hu L. (2010). Expression, purification, and characterization of recombinant lumbrokinase PI239 in *Escherichia coli*. Protein Expr. Purif..

[38] Hu Y., Meng X.L., Xu J.P., Lu W., Wang J. (2005). Cloning and expression of earthworm fibrinolytic enzyme PM_246_ in *Pichia pastoris*. Protein Expr. Purif..

[39] Ge T., Fu S.H., Xu L.H., Tang Q., Wang H.Y., Guan K.P., Liang G.D. (2007). High density fermentation and activity of a recombinant lumbrokinase (PI239) from *Pichia pastoris*. Protein Expr. Purif..

[40] Su B., Wang Z.B., Guo Y.D., Song C.C., Hu Y.C., Wang B.C., Jin J.J. (2014). Research on the method of fibrinogen-thrombin time by coagulometer for quality control of hirudo. Chin. J. Pharm. Anal..

[41] Errasti M.E., Prospitti A., Viana C.A., Gonzalez M.M., Ramos M.V., Rotelli A.E., Caffini N.O. (2016). Effects on fibrinogen, fibrin, and blood coagulation of proteolytic extracts from fruits of *Pseudananas macrodontes*, *Bromelia balansae*, and *B. hieronymi* (Bromeliaceae) in comparison with bromelain. Blood Coagul. Fibrinolysis.

[42] Mustafa A., Thornqvist P.O., Roman E., Winberg S. (2019). The aggressive spiegeldanio, carrying a mutation in the *fgfr1a* gene, has no advantage in dyadic fights with zebrafish of the AB strain. Behav. Brain Res..

[43] Shi Y.N., Shi Y.M., Yang L., Li X.C., Zhao J.H., Qu Y., Zhu H.T., Wang D., Cheng R.R., Yang C.R. (2015). Lignans and aromatic glycosides from *Piper wallichii* and their antithrombotic activities. J. Ethnopharmacol..

[44] Bern M., Kil Y.J., Becker C. (2012). Byonic: Advanced peptide and protein identification software. Curr. Protoc. Bioinform..

[45] Misconai A., Wien F., Bulyaki E., Kun J., Moussong E., Lee Y.H., Goto Y., Refregiers M., Kardos J. (2018). BeStSel: A web server for accurate protein secondary structure prediction and fold recognition from the circular dichroism spectra. Nucleic Acids Res..

[46] Lovell S.C., Davis I.W., Arendall W.B., Bakker P.D., Word J.M., Prisant M.G., Richardson J.S., Richardson D.C. (2003). Structure validation by C*α* Geometry: *Φ*, *Ψ* and C*β* deviation. Proteins Struct. Funct. Bioinform..

[47] Bosco K.H., Brasseur R. (2005). The Ramachandran plots of glycine and pre-proline. BMC Struct. Biol..

