# Supplementary material for: Bioevaluation of Pheretima vulgaris Antithrombotic Extract, PvQ, and Isolation, Identification of Six Novel PvQ-Derived Fibrinolytic Proteases
Source: Molecules. 2021 Aug 16;26(16):4946. doi: 10.3390/molecules26164946 (PMC8402109; doi:10.3390/molecules26164946)
Supplement: Supplementary file 1 [file molecules-26-04946-s001.zip › molecules-1288071-supplementary.pdf]

molecules

MDPI

Supplementary Materials

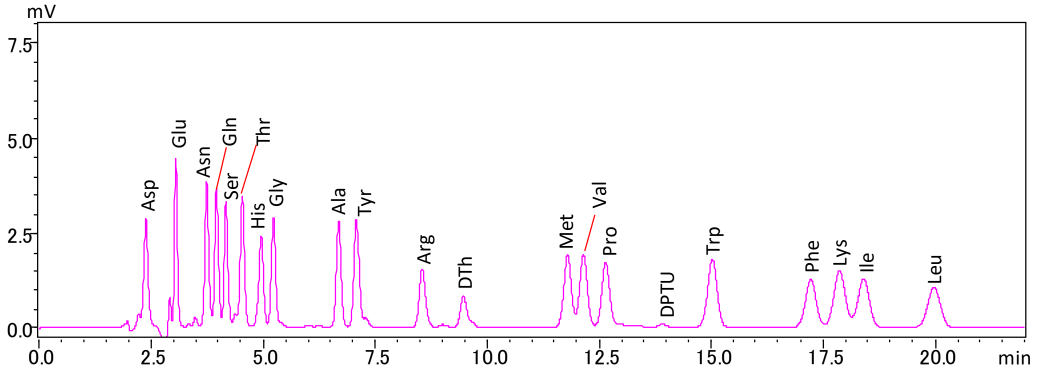

Suppl. Figure S1. Chromatography of 19 phenylthiohydantoin amino acid mixtures for *Pv*I.

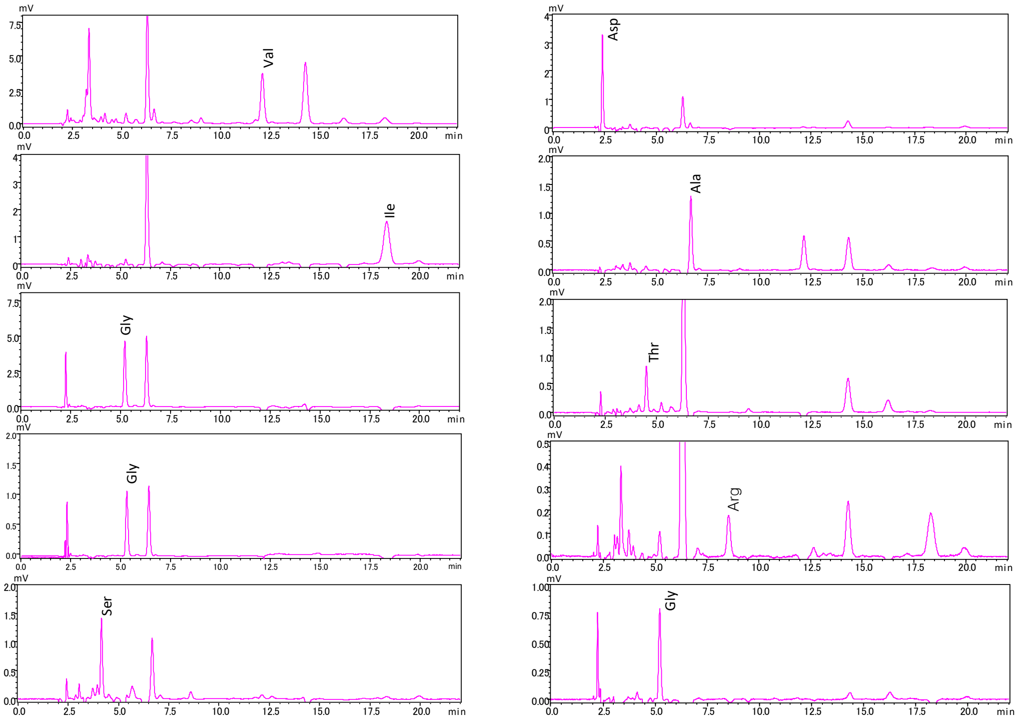

(**a**) (**b**)

Suppl. Figure S2. Chromatography of N-terminal amino acids of *Pv*I at positions 1-5 (a), 6-10 (b).

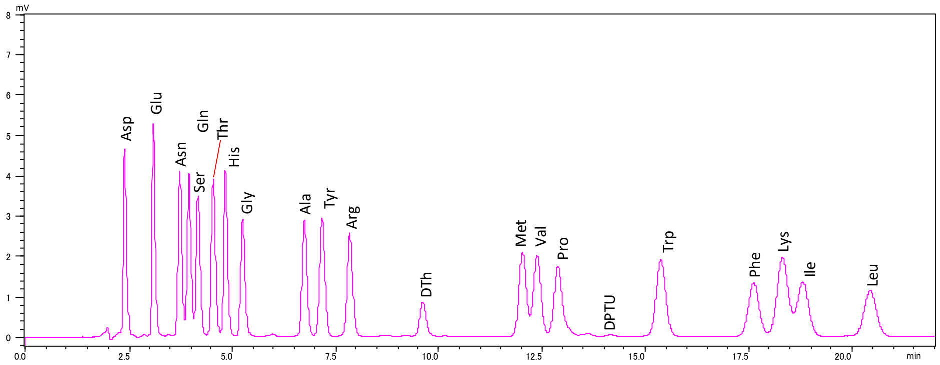

Suppl. Figure S3. Chromatography of 19 phenylthiohydantoin amino acid mixtures for *Pv*II-*Pv*VI.

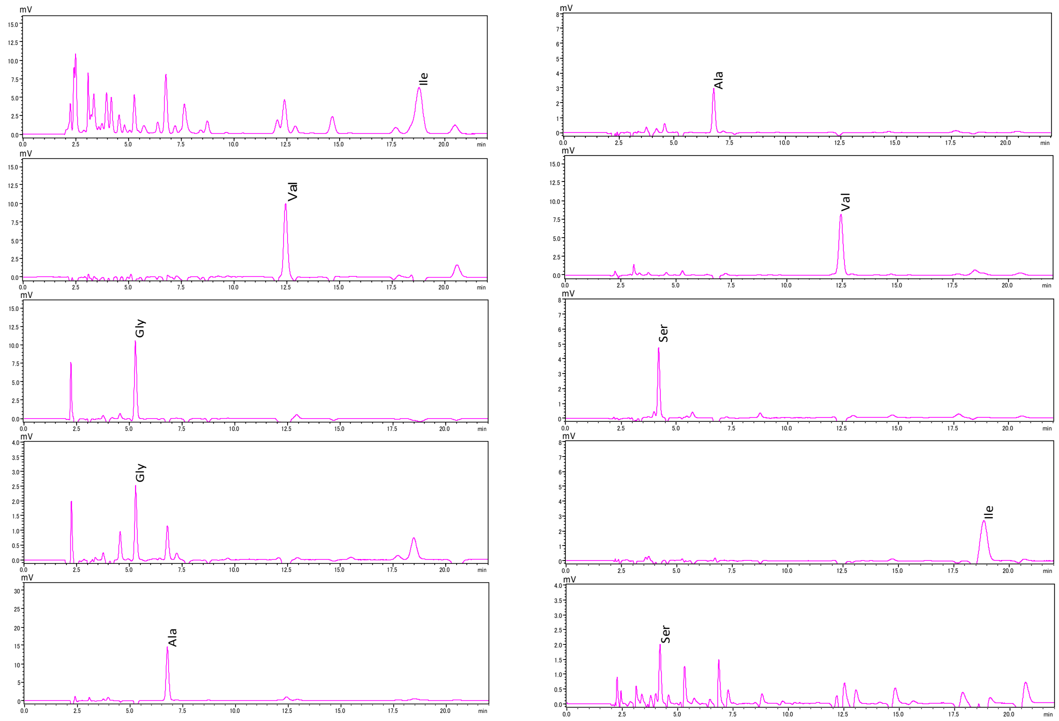

(**a**) (**b**)

Suppl. Figure S4. Chromatography of N-terminal amino acids of *Pv*II at positions 1-5 (a), 6-10 (b).

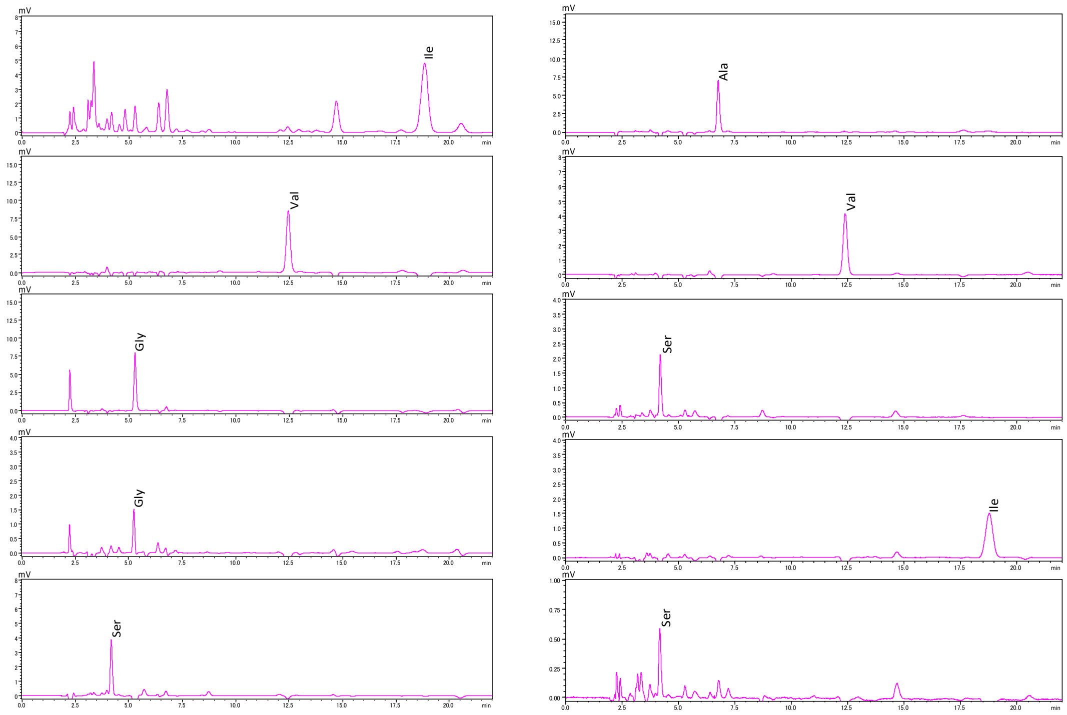

(**a**) (**b**)

Suppl. Figure S5. Chromatography of N-terminal amino acids of *Pv*III at positions 1-5 (a), 6-10 (b).

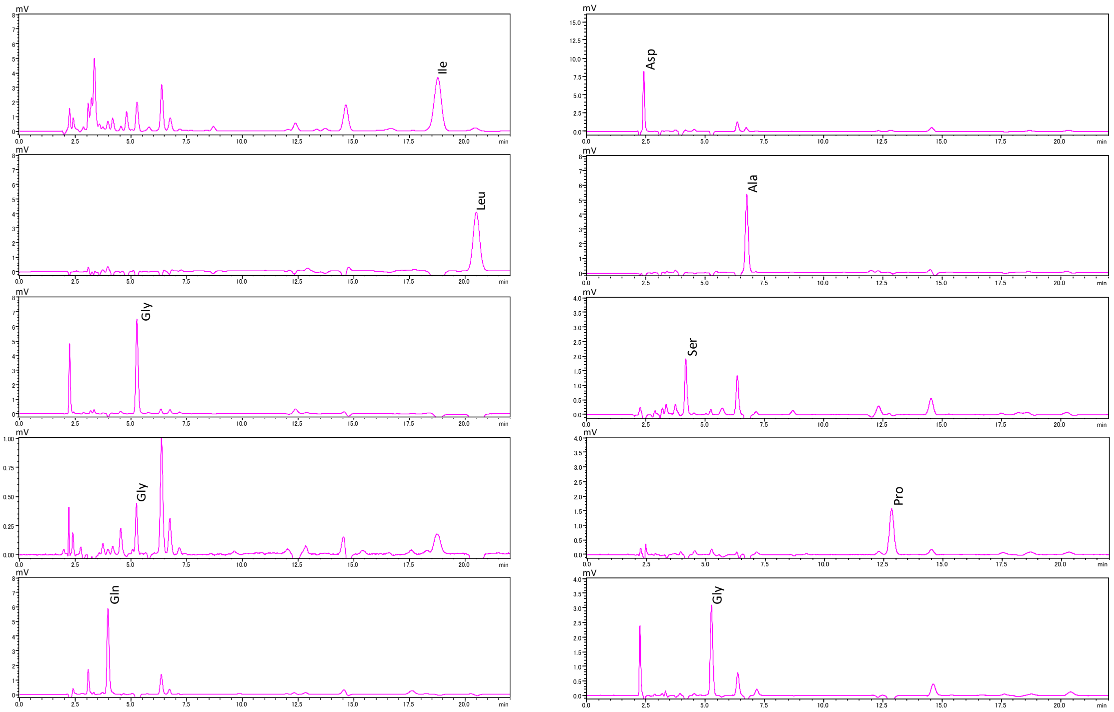

(**a**) (**b**)

Suppl. Figure S6. Chromatography of N-terminal amino acids of *Pv*IV at positions 1-5 (a), 6-10 (b).

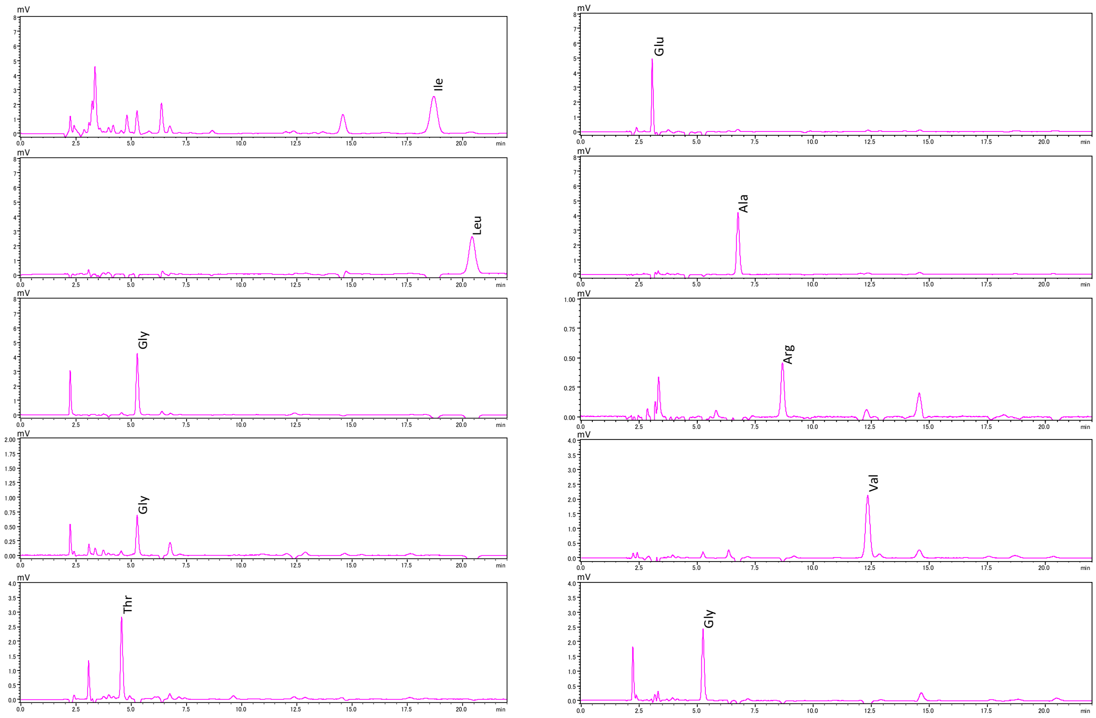

(**a**) (**b**)

Suppl. Figure S7. Chromatography of N-terminal amino acids of *Pv*V at positions 1-5 (a), 6-10 (b).

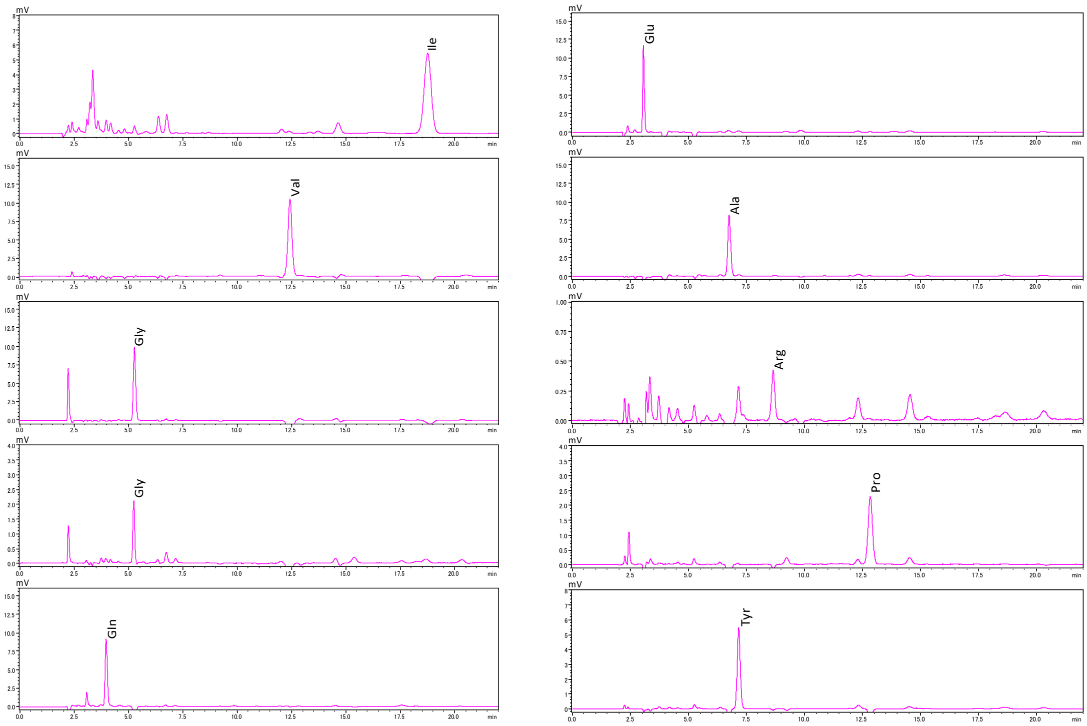

(**a**) (**b**)

Suppl. Figure S8. Chromatography of N-terminal amino acids of *Pv*VI at positions 1-5 (a), 6-10 (b).

Suppl. Table S1. The amino acids sequence of *Pv*I.

Protein name	Amino acid sequence
*Pv*I	VIGGSDATRGEFPWQLSMTRSGSHSCGATLLSSTRALCAAH<br>CVDGALTSSLAVIAGLHNRNDLTGTQS SDLSSYVMHSAYQ<br>VGSASYANDIAILHFSSVISSGGNIQTATLPANNLDDYAGTT<br>CEISGWGRTDGSNTLPNTLQ KASIPVISTAQCTVAIGGIGTIW<br>DNHICVQDPRGNSGACNGDSGGPLNCPDGVTRVVGVTSW<br>VVSSGLGDCLPSYPSVYTRVSAYLQWIADNSP

Suppl. Table S2. The amino acids sequence of *Pv*II.

Protein name	Amino acid sequence
*Pv*II	IVGGAAVSISNYPHQLSMRVAGSHSCGASLISNTRAVTAAH<br>CSGSAIGAYSMLGGTTDRTVTNCGNCQLFDLTNINIHPNYD<br>ENGNGYPNDVAVLSFISQAFNGDFQSIGLAQGSNDFAGDR<br>CTITGWGLQQQGGNLPILLQGADMTVMTNAACASEWGAL<br>SINDGHICLTDTNSGACNGDSGGPLVCGNVLAGATSWGH<br>VTCSPSFPSVY TRISYFYSWIIIQ

Suppl. Table S3. The amino acids sequence of *Pv*III.

Protein name	Amino acid sequence
*Pv*III	IVGGSAVSISDHPHQLSMRVAGSHSCGASLISNTRAVTAAH<br>CSGSAIGAYSMLGGTTDRTVTNCGNCRLFDLTNINIHPNYD<br>ENGNGYPNDVAVLSFFAQSTNGDFNTIALAQGSNDFAGDR<br>CTITGWGLQQQGG NLPILLQGADVTVMTNTACEAEWGVV<br>SVNDGHICVTDTNSGACNGDSGGPLECSGLLAGATSWGHV<br>TCSPSFPSVYTRISYFYSWIIIQ

Suppl. Table S4. The amino acids sequence of *Pv*IV.

Protein name	Amino acid sequence
*Pv*IV	ILGGQDASPGEFPWQLSQLRGGSHSCGASLLHATAALSAAH<br>CVDGALVSSVDVIAGLHQRSDLTGTQTSAAASFSIHANYDN<br>GEGTFAHDISIIHLATAIDASPANIAFLTLPPD NSYQFTGDTC<br>TLSGWGRTSASN ILPDTLQKVDIEVISTADCERLMVGVIGAD<br>CTDNHIAVHDAANNEGSCNGDSGGPMNCQLNGQTVVAG<br>ITSWGIQSGGACAPSYPSVYTRTSAYLQWISDNQ

Suppl. Table S5. The amino acids sequence of *Pv*V.

Protein name	Amino acid sequence
*Pv*V	ILGGTEARVGEIPWQLSQQRGGSHSCGASLLRPGSALSAAH<br>CVDGAPPADVRIVAGLHLRSDESTAVASLAESFLIHPSYNV<br>GEGTFPNDIAIIYLLTNINSAPVE NIDFALLPPDNVEQFVGF<br>TCVLSGWGRTSASNVLPDALQKVSIDVITTAECDSRMAAV<br>AGADCTDAHIAVFDPALQ KGSCNGDSGGPMNCPLSGEFV<br>VAGVTSWGISGGGACLPEYPSVYTRTGFYRQWIIDNIR

Suppl. Table S6. The amino acids sequence of *Pv*VI.

Protein name	Amino acid sequence
*Pv*VI	IVGGQEARPYEFPWQVSVRRRSTDSHFCGGSIINELWVVT<br>AAHCMDGESPAQVSIVAGEHDSNAISTVRESHDVAAIFV<br>HESYSSIRLTNDVSVVKVQVAFGLSVNIQPVCAPDPANLY<br>THQKSQCSGWGSLSSGGACCPPTLRYVTLNITTNAFCDQI<br>YTTDDITDDMICATDNTGMTDRDSCQGDSGGPLTVKDGN<br>GI FSLVGIVSWGIGCASGWPGVYARVGYQAGWITNIINNN

Suppl. Table S7. The gene sequence of *Pv*I.

Protein name	Gene sequence
*Pv*I	GTCATCGGTGGATCGGATGCCACCCGCGGAGAGTTCCC<br>GTGGCAGCTGTCGATGACACGCAGCGGTTCACACAGCT<br>GCGGAGCAACGCTGCTGAGTTCGACGCGCGCTCTCTGC<br>GCCGCTCACTGCGTCGACGGAGCACTAACATCCAGCCT<br>CGCAGTCATTGCTGGTCTTCATAATCGCAACGACTTAAC<br>AGGAACTCAGAGCTCCGACCTTTCCAGCTATGTTATGCA<br>CTCAGCCTACCAGGTTGGCAGCGCGTCCTATGCAAACG<br>ACATTGCCATTCTGCATTTCTCTTCTGTCATCAGCAGCG<br>GTGGAAATATTCAGACCGCCACTCTTCCAGCAAATAAC<br>CTCGACGACTATGCCGGTACCACATGTGAAATATCTGG<br>CTGGGGCCGCACAGACGGAAGCAACACTCTTCCAAAC<br>ACCCTTCAGAAGGCCTCCATTCCAGTCATATCTACAGC<br>TCAATGCACTGTAGCCATTGGTGGAATAGGAACGATCT<br>GGGATAACCACATCTGCGTCCAAGACCCTAGAGGAAA<br>TAGTGGAGCTTGCAATGGTGACAGTGGTGGTCCGCTCA<br>ATTGCCCAGATGGCGTTACCCGAGTCGTGGGTGTTACT<br>TCTTGGGTTGTTTCGAGCGGTCTCGGTGACTGCCTTCCG<br>TCTTATCCCTCGGTCTACACGCGTGTGAGCGCCTACTTG<br>CAATGGATCGCTGACAACAGTCCCTAA

Suppl. Table S8. The gene sequence of *Pv*II.

Protein name	Gene sequence
*Pv*II	ATCGTCGGTGGAGCAGCCGTCAGCATCAGCAACTATCC<br>GCATCAGCTGTCGATGAGGGTAGCGGGATCTCATTCGT<br>GCGGAGCGTCCCTCATTTCAAACACCAGAGCCGTCACC<br>GCTGCTCACTGCAGTGGATCCGCAATCGGAGCTTATTC<br>CATGCTGGGAGGCACTACCGACCGAACAGTCACCAAC<br>TGCGGCAACTGTCAACTCTTTGACTTGACAAACATCAA<br>TAT TCATCCTAACTACGATGAGAATGGAAATGGATAC<br>CCCAACGATGTGGCTGTTCTCAGCTTTATTTCACAGGC<br>CTTCAACGGCGACTTCCAGTCCATTGGTTTGGCTCAAG<br>GGTCAAACGATTTTGCAGGAGACAGATGTACCATCAC<br>TGGCTGGGGTCTGCAACAACAAGGTGGAAATCTGCCA<br>ATCCTATTGCAGGGAGCAGATATGACTGTTATGACTA<br>ACGCTGCTTGCGCGTCTGAATGGGGTGCTCTCAGTATA<br>AACGATGGACACATTTGTCTCACAGATACCAACTCCG<br>GCGCTTGCAACGGGGACAGTGGTGGTCCACTGGTGTG<br>CGGTAATGTTCTTGCTGGAGCGACGTCCTGGGGTCAT<br>GTGACATGCAGCCCGTCGTTTCCTTCCGTCTACACGAG<br>AATCAGCTACTTCTATTCCTGGATCATCATTCAATAA

Suppl. Table S9. The gene sequence of *Pv*III.

Protein name	Gene sequence
*Pv*III	ATTGTTGGAGGATCGGCCGTCAGCATAAGCGACCATCC<br>GCACCAGCTGTCGATGAGGGTAGCGGGATCTCATTCGT<br>GCGGAGCGTCCCTCATTTCAAACACCAGAGCCGTCACC<br>GCTGCTCACTGCAGTGGATCCGCAATCGGAGCTTATTC<br>CATGCTGGGAGGCACTACCGACCGAACAGTCACCAAC<br>TGCGGCAACTGTCGACTCTTTGACTTGACAAACATCAA<br>TATTCATCCTAACTACGATGAGAATGGAAATGGATACC<br>CCAACGATGTGGCTGTTCTTAGCTTTTTTGCACAGTCCA<br>CCAACGGTGACTTCAATACCATTGCTTTGGCTCAAGGG<br>TCAAACGACTTTGCAGGAGACAGATGTACCATCACTG<br>GCTGGGGTCTGCAACAACAAGGTGGAAATCTGCCAAT<br>CCTATTGCAGGGAGCAGATGTGACTGTTATGACTAACA<br>CTGCTTGCGAGGCGGAATGGGGTGTTGTCAGTGTAAAC<br>GATGGACACATTTGTGTCACAGATACCAACTCCGGCGC<br>TTGCAACGGAGACAGTGGTGGTCCATTGGAGTGCAGTG<br>GTCTTCTCGCTGGAGCGACGTCCTGGGGTCATGTGACAT<br>GCAGCCCGTCGTTTCCATCCGTCTACACGAGAATCAGC<br>TACTTCTATTCCTGGATCATCATTCAATAA

Suppl. Table S10. The gene sequence of *Pv*IV.

Protein name	Gene sequence
*Pv*IV	ATTCTCGGAGGACAGGATGCCTCTCCTGGAGAGTTCCC<br>GTGGCAACTGTCCCAGTTGAGAGGTGGAAGCCACAGC<br>TGCGGTGCCTCTCTCCTCCACGCCACAGCTGCTCTGAG<br>CGCCGCCCACTGCGTCGACGGAGCACTCGTATCATCAG<br>TCGATGTCATCGCCGGTCTTCATCAGCGCTCAGATCTTA

	CTGGAACACAGACTTCAGCTGCTGCGTCATTCAGCATT<br>CACGCGAACTACGATAACGGAGAGGGCACATTCGCAC<br>ATGATATCTCCATCATCCACCTCGCCACAGCCATCGAC<br>GCCTCGCCAGCCAACATCGCGTTTTGACCCTTCCTCCA<br>GACAACAGCTATCAGTTTACTGGTGACACCTGCACTCT<br>CAGTGGATGGGGACGCACATCTGCCAGCAACATTCTTC<br>CGGACACACTGCAGAAAGTTGACATCGAAGTCATCAG<br>CACAGCCGATTGCGAACGCCTTATGGTTGGCGTCATTG<br>GTGCGGACTGCACTGATAATCATATCGCAGTTCACGAT<br>GCTGCCAATAACGAGGGATCGTGCAACGGTGACAGCG<br>GTGGTCCGATGAACTGCCAACTCAATGGTCAGACTGTG<br>GTTGCCGGAATTACGTCGTGGGGAATCCAGTCGGGCGG<br>CGCCTGTGCACCATCCTACCCATCTGTCTACACCAGGAC<br>TTCAGCCTACCTTCAATGGATCAGCGACAACCAATAG

Suppl. Table S11. The gene sequence of *Pv*V.

Protein name	Gene sequence
*Pv*V	ATCCTTGGAGGAACGGAAGCCAGAGTTGGAGAGATCC<br>CATGGCAGCTGTCGCAGCAGAGAGGCGGAAGTCACAG<br>CTGCGGAGCGTCTCTTCTCAGGCCCGGTTCGGCCCTCAG<br>CGCCGCTCACTGCGTTGACGGAGCACCGCCAGCAGATG<br>TGCGAATTGTCGCTGGACTTCATTTGCGCTCAGATGAAT<br>CTACTGCAGTGGCTTCCCTTGCTGAGAGTTTCCTAATTC<br>ACCCGAGTTACAACGTTGGAGAAGGAACTTTCCCAAAC<br>GACATCGCCATCATCTACCTATTAACAAATATCAACTCT<br>GCTCCAGTAGAAAACATTGATTTTGCTCTTCTACCTCCA<br>GACAACGTCGAGCAATTCGTCGGATTTACTTGCGTGCTC<br>AGTGGATGGGGACGCACATCGGCCAGCAATGTACTTCC<br>CGATGCCCTGCAGAAGGTCAGCATCGACGTCATCACCA<br>CAGCCGAATGCGACTCACGCATGGCCGCTGTTGCTGGA<br>GCCGACTGCACTGATGCTCACATCGCCGTCTTCGATCCC<br>GCTTTGCAGAAAGGATCGTGCAACGGTGACAGCGGTGG<br>CCCAATGAACTGCCCTCTGAGCGGTGAATTTGTGGTTGC<br>TGGTGTGACGTCATGGGGAATTTCGGGAGGCGGTGCCT<br>GTCTGCCAGAATACCCATCAGTCTACACCAGAACTGGA<br>TTCTACCGTCAATGGATCATTGACAACATTCGCTAG

Suppl. Table S12. The gene sequence of *Pv*VI.

Protein name	Gene sequence
*Pv*VI	ATCGTCGGAGGGCAGGAAGCCAGACCATACGAATTCC<br>CGTGGCAGGTGTCCGTAAGGCGCAGGTCCACTGACAG<br>CCATTTCTGCGGCGGCAGCATCATCAATGAACTCTGGG<br>TTGTTACAGCTGCTCACTGCATGGACGGAGAGAGTCCC<br>GCGCAGGTGTCGATCGTCGCAGGGGAACACGACAGCA<br>ACGCCATCAGCACCGTTCGCGAGAGCCATGACGTCGC<br>CGCCATCTTCGTGCACGAGTCCTACAGCAGCATCCGAC<br>TGACAAACGACGTGTCCGTCGTCAAGGTGCAGGTGGC<br>CTTCGGCTTGTCCGTGAACATCCAGCCGGTCTGCGCAC<br>CGGACCCGGCCAACCTCTACACGCACCAGAAGAGCCA

	GTGCTCCGGTTGGGGAAGCCTCAGTTCAGGCGGAGCTT<br>GCTGCCCACCCACCTTGCGTTACGTTACCCTCAACATC<br>ACCACGAATGCCTTCTGTGACCAGATCTACACCACCGA<br>TGACATCACGGACGACATGATCTGCGCCACAGATAAC<br>ACTGGAATGACCGATAGAGATTCGTGCCAGGGTGATT<br>CCGGTGGCCCCCTGACCGTCAAGGACGGAAACGGCAT<br>CTTCTCCCTCGTCGGTATCGTCTCGTGGGGTATCGGCTG<br>TGCCTCCGGATGGCCGGGAGTCTACGCCAGGGTGGGTT<br>ATCAAGCAGGCTGGATCACGAACATCATTAACAACAAC<br>TGA
